# Applications of Functional Near-Infrared Spectroscopy (fNIRS) Neuroimaging in Exercise–Cognition Science: A Systematic, Methodology-Focused Review

**DOI:** 10.3390/jcm7120466

**Published:** 2018-11-22

**Authors:** Fabian Herold, Patrick Wiegel, Felix Scholkmann, Notger G. Müller

**Affiliations:** 1 Research Group Neuroprotection, German Center for Neurodegenerative Diseases (DZNE), Magdeburg 39120, Germany; notger.mueller@dzne.de; 2 Department of Sport Science, University of Freiburg, Freiburg 79117, Germany; patrick.wiegel@sport.uni-freiburg.de; 3 Bernstein Center Freiburg, University of Freiburg, Freiburg 79104, Germany; 4 Biomedical Optics Research Laboratory, Department of Neonatology, University Hospital Zurich, University of Zürich, Zürich 8091, Switzerland; felix.scholkmann@usz.ch; 5 Center for Behavioral Brain Sciences (CBBS), Magdeburg 39118, Germany; 6 Department of Neurology, Medical Faculty, Otto von Guericke University, Magdeburg 39120, Germany

**Keywords:** fNIRS, optical imaging, physical activity, cognition, executive functions, working memory

## Abstract

For cognitive processes to function well, it is essential that the brain is optimally supplied with oxygen and blood. In recent years, evidence has emerged suggesting that cerebral oxygenation and hemodynamics can be modified with physical activity. To better understand the relationship between cerebral oxygenation/hemodynamics, physical activity, and cognition, the application of state-of-the art neuroimaging tools is essential. Functional near-infrared spectroscopy (fNIRS) is such a neuroimaging tool especially suitable to investigate the effects of physical activity/exercises on cerebral oxygenation and hemodynamics due to its capability to quantify changes in the concentration of oxygenated hemoglobin (oxyHb) and deoxygenated hemoglobin (deoxyHb) non-invasively in the human brain. However, currently there is no clear standardized procedure regarding the application, data processing, and data analysis of fNIRS, and there is a large heterogeneity regarding how fNIRS is applied in the field of exercise–cognition science. Therefore, this review aims to summarize the current methodological knowledge about fNIRS application in studies measuring the cortical hemodynamic responses during cognitive testing (i) prior and after different physical activities interventions, and (ii) in cross-sectional studies accounting for the physical fitness level of their participants. Based on the review of the methodology of 35 as relevant considered publications, we outline recommendations for future fNIRS studies in the field of exercise–cognition science.

## 1. Introduction

Availability of oxygen is crucial for cognitive processes to be intact [1,2,3,4] and a lack of oxygen in the brain leads to lower cognitive performance [1,5]. Emerging evidence suggests that oxygen availability can be enhanced by physical activity. For example, an acute bout of physical activity increases cognitive performance and is accompanied by higher levels of oxygenated hemoglobin in the prefrontal areas of the human brain [6,7,8,9]. A similar relationship was noticed in cross-sectional studies, which found that more hours of weekly physical activity [10] and higher cardiorespiratory fitness levels [11,12,13] are associated with higher cerebral oxygenation levels and superior cognitive performance. However, since physical-activity-induced neurobiological mechanisms (e.g., cerebral oxygen availability), which may contribute to improved cognitive performance are not fully understood yet [14,15,16], it seems helpful to apply state-of-the-art neuroimaging methods in order to foster our understanding of the effects of physical activity on cognition [17,18]. Based on the crucial role of oxygen availability for cognition together with findings suggesting that physical activity positively influences oxygen availability and cognitive performance, neuroimaging tools that can quantify tissue oxygenation (metabolism) and hemodynamics (blood flow) seem especially suitable to answer emerging research questions in the field of exercise–cognition science (for review of emerging research questions please see References [17,18,19,20]). While cerebral oxygenation and hemodynamics can be quantified with functional magnetic resonance imaging (fMRI), positron-emission-tomography (PET) and functional near-infrared spectroscopy (fNIRS) [21,22,23,24], electroencephalography (EEG) is a frequently used electrophysiological technique to record the electric signals of the brain [25,26,27,28]. However, all mentioned neuroimaging techniques have unique methodological advantages and disadvantages that have to be traded off with regard to the intended research purpose.

fMRI is often considered as the gold standard for the assessment of brain activity as it offers the advantage to measure functional changes across the whole brain with a high spatial resolution (e.g., <4.0 mm) [29,30,31,32,33]. However, fMRI acquisition costs are relatively high, fMRI is susceptible to movement artefacts (e.g., requires rigorous head stabilization), fMRI is relatively noisy during the measurements, fMRI provides a relative low temporal resolution (e.g., ≈0.5 Hz), and fMRI cannot be used in special cohorts (e.g., individuals with metallic implants or claustrophobia) [29,30,32,34,35,36]. PET allows the assessment of changes in various substances (e.g., glucose), but PET scans are relatively expensive and repeated measurements within short time intervals are ethically not feasible due to the use of radioactive tracer substances [22,31]. EEG, which measures the brain activation directly and non-invasively based on neuroelectric signals of neurons [37], offers a high temporal resolution (e.g., >1000 Hz) but suffers from a relatively weak spatial resolution (e.g., ≈5.0–9.0 cm) [27,29,30,38,39,40,41]. Furthermore, EEG is relative susceptible to artefacts (e.g., due to sweat or muscle activity), is time consuming in preparation (e.g., when gel is used), and the obtained signals are hard to interpret for non-experts [27,29,38,42,43]. Hence, fMRI, PET, and EEG have specific restrictions that hamper their efficient utilization in exercise–cognition settings (e.g., after an acute bout of physical activity).

**Figure 1:**
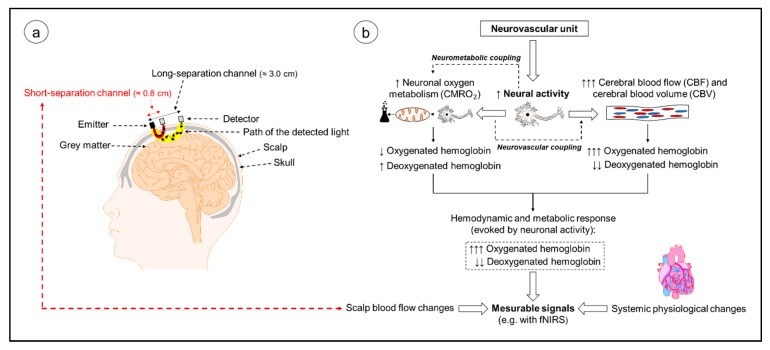
(**a**) Schematic illustration of the neurovascular unit and the changes in cerebral hemodynamics and oxygenation induced by neural activity. (**b**) Exemplary illustration of a possible NIRS montage on the human head and the assumed banana-shaped course of detected light of “short-separation channels” and of “long-separation channels”. fNIRS, functional near-infrared spectroscopy; CMRO_2_, cerebral metabolic rate of oxygen; ↑, increase; ↓, decrease.

**Figure 2:**
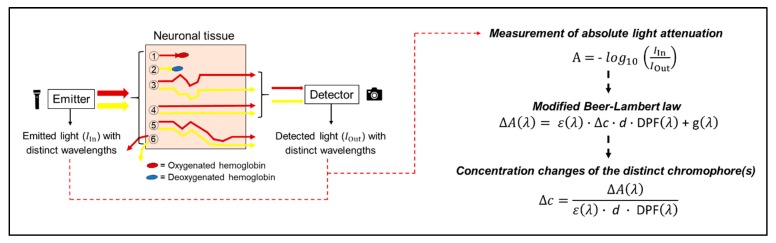
Schematic illustration of light propagation through the neuronal tissue. On the left side of the illustration, possible photon paths for different wavelengths are depicted (red colors represent wavelengths of λ > 800 nm (mainly absorbed by oxyHb—see Photon 1), whereas yellow colors represent wavelengths of λ < 800 nm (mainly absorbed by deoxyHb—see Photon 2). Path 3 represents a photon that undergoes some scattering events before being recorded by a detector. Path 4 represents a ballistic photon. Path 5 represents a photon that, after some scattering events, is not recorded by a detector (lost due to forward scattering). Path 6 represents a photon that is lost due to backward scattering. In the right part of the illustration, the formulas to calculate concentration changes in chromophores are shown (based on continuous-wave NIRS). The symbols have the following meanings: A: light attenuation, or ΔΑ(λ): changes in light attenuation at a certain wavelength (λ); *Ι_Ιn_*: intensity of emitted light; *Ι_Out_*: intensity of recorded light; ε(λ): the extinction coefficient of the chromophore at a certain wavelength (λ); Δc: changes in chromophore concentration; d: separation (distance) between source and detector; DPF(λ): differential path length factor (DPF) for a certain wavelength (λ); g(λ): scattering at a certain wavelength (λ), where g is cancelled out since it is assumed to be negligible when only light attenuation (as in continuous-wave NIRS) is considered [45,54,58].

fNIRS is an optical neuroimaging technique that is based on the theory of neurovascular coupling and optical spectroscopy (see Figure 1a,b and Figure 2) [44,45]. As shown in Figure 1b, an increase in neural activity causes an increase in the oxygen metabolism, which is necessary to satisfy energetic demands of the neuronal tissue (neurometabolic coupling) [40,46,47]. Within the neuronal oxygen metabolism, oxygen is consumed to produce energy, leading to a decrease in the concentration of oxyHb and to an increase in the concentration of deoxyHb [46,47,48]. Neural activity triggers local changes in cerebral hemodynamics that induce an intensified blood flow to the activated brain regions (neurovascular coupling) [40,46,49,50]. Since the local supply of oxygen is greater than its consumption, in activated brain regions, a higher concentration of oxyHb and a decreased concentration of deoxyHb is to be observed (see Figure 1b) [40,47].

When applying fNIRS, light with different wavelengths in the near-infrared spectrum is emitted by a source on the scalp and after the travelling through different layers (skull, cerebrospinal fluid), this light reaches neuronal tissue [39,40,51]. Inside the tissue, the light undergoes absorption and scattering that contributes to light attenuation [51,52,53]. During absorption, the energy of the photons is transformed into internal energy of the respective medium (see Figure 2, Photon 1 and 2) [40]. Scattering forced the photons to deviate from their initially straight trajectories and increase the length of their travelled paths (see Figure 2, Photons 3 and 4) [40,52]. The non-absorbed components of the scattered light can be measured by a detector placed on the head’s surface (e.g., see Figure 1a) [39,51]. Based on the activity-dependent regional increase of oxyHb and decrease of deoxyHb, the light absorption rate of the neuronal tissue in the activated brain region changes and influences, in turn, light attenuation [40,51,54]. The regional changes in light absorption as a function of neuronal activity and the different light absorption spectra of the chromophores (e.g., λ > 800 nm mainly oxyHb, λ < 800 nm mainly deoxyHb) allow for the non-invasive quantification of local changes in cortical oxyHb and deoxyHb concentration via the modified Beer–Lambert law [39,40,51]. The cortical concentration changes in oxyHb and deoxyHb are used as an indirect indicator of regional brain activation (such as in functional magnetic resonance imaging) [36,39,54]. The basic principles of fNIRS are summarized in Figure 1 and Figure 2. We will focus on the description of continuous-wave fNIRS because commercially available fNIRS devices are mainly based on the continuous-wave technology [40,47]. In continuous-wave fNIRS the absolute changes in the attenuation coefficient are determined (e.g., difference between the intensity of the emitted light and detector-determined light intensity; see Figure 2). Thus, the fNIRS signals obtained reflect relative concentration changes (e.g., relative to the first measured values) [47,49,55,56,57]. A detailed description about other types of NIRS devices is given in the Supplementary Material.

fNIRS provides some advantages that make it well-situated to investigate the effects of physical activity on cognitive performance and cerebral oxygenation/hemodynamics. Advantages of fNIRS compared to other neuroimaging techniques (e.g., fMRI and PET) are: non-invasiveness, a relatively good spatial (≈1.0–3.0 cm) and temporal resolution (normally up to 10 Hz), portability, a low noise level during operation, relative low acquisition costs, robustness against motion artefacts that make a strict immobilization or sedation of participants unnecessary, the possibility to investigate cortical activity in individuals with metallic implants or claustrophobia, and the opportunity to conduct repeated measures in short time intervals (since no radioactive tracer substance as in PET is used) [21,22,29,30,32,34,38,40,41,52,54,59]. The mentioned advantages make fNIRS eminently suitable for application in special cohorts such as children [29,34] or neurological patients [23,60,61]. Furthermore, while fMRI mainly relies on the paramagnetic properties of deoxyHb, fNIRS can be used to quantify both changes of deoxyHb and of oxyHb [53,54,58,59,62]. The simultaneous assessment of deoxyHb and oxyHb allows the quantification of further markers of cortical activation such as tissue oxygenation (TOI: oxyHb concentration/total hemoglobin concentration) and cortical hemodynamics (blood volume, total hemoglobin concentration (totHb)) [22,30,54,58]. Moreover, fNIRS is even capable of evaluating changes in cytochrome oxidase levels, which constitute a marker of the cellular oxygen metabolism [63,64,65,66,67]. On the downside, fNIRS is limited to cortical layers [32,40,52] because the penetration depth is, in general, less than half of the source-detector separation [54,62,68]. Furthermore, fNIRS suffers from its vulnerability to changes in scalp blood flow and to changes in systemic physiology (e.g., increase in heart rate) [30,32,40,69,70,71,72]. Of note, while fNIRS has proven to be a useful and reliable tool in some research fields (e.g., motor control) [25,36,73], currently no standardized procedures regarding the processing of fNIRS data are available [21,30,38,41,74]. Moreover, the methods used to measure cortical hemodynamics during cognitive tasks are diverse [74]. There is no consensus yet regarding a standardized methodology (e.g., application, processing and analysis) that limits the comparability across studies because numerous parameters vary in the (pre-)processing and analysis algorithms (e.g., value of differential path length factor, filter cut-off frequencies). While first attempts were undertaken to standardize the application, processing, and analysis of fNIRS in other research areas (e.g., motor control) [36,41], in the field of exercise–cognition science, so far only systematic reviews summarizing the findings but not the methodology of fNIRS are available [52,75]. Moreover, a systematic review pooling fNIRS studies investigating the influence of physical activities (e.g., 10 min of cycling) or assessing the influence of habitual physical activity on the performance of standardized cognitive tests and the corresponding cortical hemodynamic responses is completely lacking. Since (i) the recommendations of previously methodologically focused reviews [36,41] are not fully transferable to the field of exercise–cognition (e.g., due to differences in biasing factors such as the influence of physiological artefacts on temporal delay between being physically active and cognitive testing), and (ii) the great interest from various scientific disciplines in the relationship between physical activity, central nervous system, and cognition (e.g., sport science, neuroscience, psychology), this systematic review aims to summarize the methodological details and findings of studies investigating the influence of physical activity on cognition while measuring cortical hemodynamics with fNIRS. Based on the results of this systematic literature survey, we derive recommendations for future studies.

## 2. Methods

### 2.1. Search Strategy and Process

On the 13 October 2018, two independent researchers performed a systematic literature search in seven electronic databases to identify all relevant studies employing fNIRS to measure cortical hemodynamics during a standardized cognitive task (i) prior and after a single bout of physical activities and/or long-term physical exercise programs (>two exercise sessions), and (ii) linking cortical hemodynamics to measures of physical activity or physical fitness (e.g., cardiorespiratory fitness) [76]. In all databases, the following search strings were used:

- exercis* OR fitness OR physical activity OR training OR strength OR endurance OR aerobic OR agility OR cycling OR running OR dance OR dancing OR walking OR “going outdoor”
- cogniti* OR mental OR executive OR memory OR attention OR “reaction time” OR “response time” OR processing OR Stroop OR Flanker OR Sternberg OR “Verbal Fluency Task” OR “Tower of Hanoi” OR “Tower of London” OR “Wisconsin card sorting task” OR “Trail Making Test” OR “visual search” OR visuospatial OR “decision making” OR oddball OR accuracy OR error
- NIR OR fNIR* OR "functional near-infrared spectroscopy" OR "near-infrared spectroscopy" OR "functional near-infrared spectroscopic" OR "optical imaging system" OR "optical topography" OR oxygenation

In PubMed, PsycInfo, CINAHL, and Sportdiscus, no restriction was applied. In Cochrane Library, we limited the search to “trials,” in Web of Science to “topic,” and in Scopus to “title, abstract, keywords.” We identified and added four relevant studies [10,77,78,79] after screening of the references of the included studies and after searching for further studies of the included workgroups.

**Figure 3:**
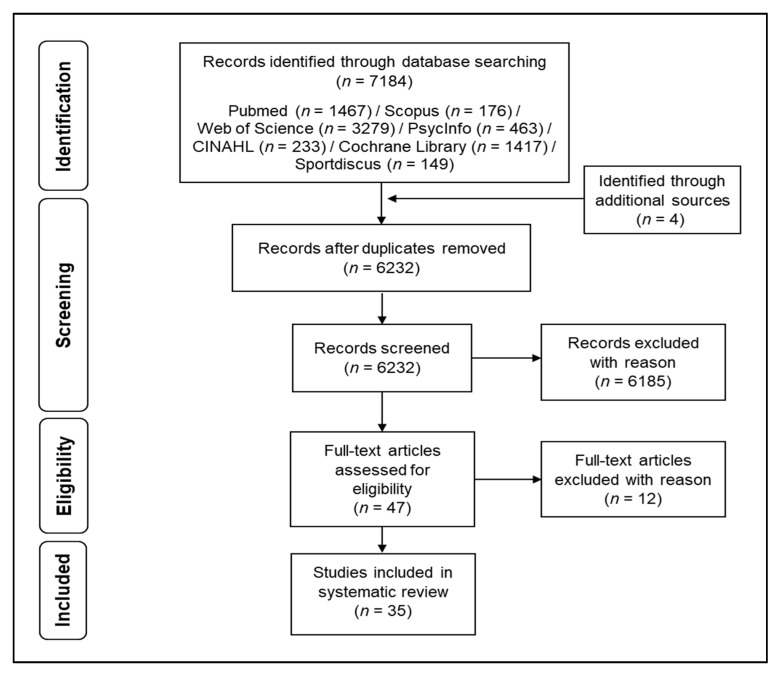
Flow chart with information about the search, screening, and selection processes, which led to the identification of relevant articles included in this review.

Afterwards, the results of the systematic search were loaded in a citation manager, which was used for further analyses and for removing of the duplicates (see Figure 3).

### 2.2. Inclusion and Exclusion Criteria

Screening for relevant studies was conducted according to the PICOS-principle [76,80]. PICOS stands for participants (P), intervention (I), comparisons (C), outcomes (O), and study design (S) [76,80]. All age groups, regardless of pathology, were included given that brain activity had been measured with fNIRS during a cognitive test prior and after a physical intervention. Furthermore, cross-sectional studies were included when they had assessed the physical activity level (e.g., via questionnaire) or the physical fitness level (e.g., cardiorespiratory fitness level) and conducted cognitive testing while measuring the cortical hemodynamics with fNIRS. Studies written in a non-English language [81,82,83], conducted by performing the cognitive tests without measuring brain activation with fNIRS [84,85,86,87,88,89], measuring brain activation during the physical exercises [88,89,90,91], and those with a focus on the effect of nutritional supplement on cognitive performance [92] were excluded from the present literature survey.

### 2.3. Data Extraction

From the 35 studies considered to be relevant, we extracted information about first author, year of publication, population characteristics including age, gender, health status, cardiorespiratory fitness level, exercise characteristics (e.g., intensity, duration, type of exercise), and cognitive testing (e.g., tested cognitive domain, administration after exercise cessation). Furthermore, information about fNIRS methodology regarding optode placement, source-detector separation, differential path length factor (DPF), the applied filter methods, the data processing procedures, data analysis (e.g., markers of cortical activation), and the main findings were extracted. When articles provided an incomplete description of their methodology, we contacted the authors via e-mail.

Please note that in this review a single session of physical exercise is referred as “physical activity” rather than ”physical exercise” because “exercise” is per definition a structured and planned form of physical activity that is intended to improve or maintain a distinct fitness level [17,93,94,95].

## 3. Results

In the following section, information about methodological approaches (e.g., recording, processing, and analysis of fNIRS data) and findings of the 35 reviewed studies are provided.

### 3.1. fNIRS Optode Placement

**Figure 4:**
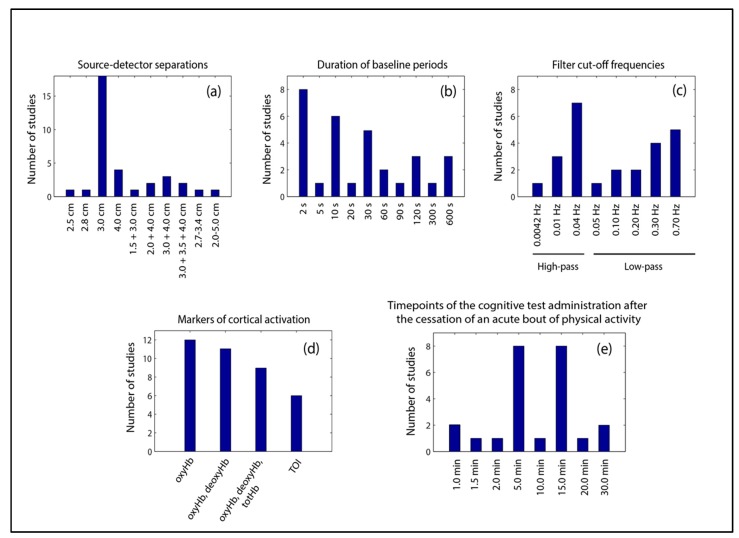
Overview on (**a**) source-detector separations, (**b**) durations of baseline periods, (**c**) filter cut-off frequencies, (**d**) markers of cortical activation, and (**e**) timepoints of the cognitive test administration after the cessation of an acute bout of physical activity. cm: centimeters; deoxyHb: deoxygenated hemoglobin; Hz: Hertz; min: minutes; oxyHb: oxygenated hemoglobin; s: seconds; TOI: tissue oxygenation index; totHb: total hemoglobin.

The majority of the reviewed studies used the international EEG system for the placement of the optodes [6,7,8,11,12,13,77,79,96,97,98,99,100,101,102,103,104,105,106,107,108,109] and set the source-detector separations at 3.0 cm [6,7,8,12,77,78,79,96,99,100,102,104,106,107,109,110,111]. A detailed overview about the used source-detector separations utilized in the remaining studies is given in Figure 4a. In two studies, individual fMRI-scans [111,112], and in seven studies, virtual registration (e.g., using 3-D digitizer), was performed [6,7,8,12,109,113,114]. In almost all reviewed studies, the optodes were placed over distinct parts of the prefrontal cortex. A detailed overview is provided in Table 1.

### 3.2. fNIRS Experimental Paradigms of Data Recording

In almost all studies baseline brain activation was assessed in a sitting position [6,7,8,11,12,13,77,78,79,96,97,98,99,100,101,102,103,104,105,106,107,108,109,110,111,115,116,117,118,119,120]. The quantification of baseline brain activation lasted from 2 s [6,7,8,12,77,106,109] to 10 min [97,116,119]. Other commonly used durations for the evaluation of baseline brain activation were 10 s [13,79,99,100,107], 30 s [102,104,105,108,120], or 120 s [103,111,118]. An overview about baseline durations is provided in Figure 4b.

A block design was used in eleven studies [11,13,78,99,100,102,103,104,107,112,113], whereas an event-related design was applied in ten studies [6,7,8,12,77,106,109,110,111,114]. In the remaining studies, cognitive testing was performed after the assessment of baseline brain activity [79,96,97,98,101,105,108,115,116,117,118,119,120].

### 3.3. DPF Values

The differential path length factor (DPF) is a dimensionless correction factor that accounts for the increase in the optical pathlength caused by the scattering of light in biological tissue and is multiplied with the source-detector separation to estimate the “true” path length that light has travelled [12,121,122]. A constant wavelength-independent DPF was used in six studies [11,97,101,105,118,119]. In those six studies, DPF values of 4.0 [97,105,119], 5.9 [101], and 5.93 [11,118] were applied. A constant, wavelength-dependent DPF with the values of 7.25/6.38 (760 nm/850 nm) was used in two studies [103,110]. Two studies [108,120] used age-dependent DPF values calculated as described in Duncan et al. [123]. In the remaining studies, arbitrary units [6,7,8,12,13,77,78,79,96,100,102,104,106,107,109,111] or saturation index/tissue oxygenation index (*StO_2_* or TOI = oxyHb/totHb) [10,105,108,115,116,120], which do not rely on specific DPF values [124,125], were used.

### 3.4. fNIRS Signal Filtering

In three studies the filtering of fNIRS signals was conducted using a low-pass filer [13,100,107] or a high-pass filter [11,97,116]. In eight studies a bandpass filter, which consists of low-pass filter and high-pass filter, was used [6,7,8,12,96,103,109,110]. The cut-off frequencies of low-pass filters and high-pass filters are shown in Figure 4c. In addition to low-pass filters, high-pass filters, bandpass filters, or—in one study each—filter methods based on principal component analysis (PCA) [11], Gaussian smoothing [119], or moving averages [79] were applied. In two studies, spike artefact removal [103,110] was conducted, and in four studies, signals from short-separation channels were used to correct for superficial artefacts [105,113,116,120].

### 3.5. Final fNIRS Data Processing

In almost all of the studies reviewed, a baseline correction [6,7,8,11,12,13,77,78,79,97,98,99,100,101,102,103,105,106,107,108,109,111,117,118,119,120] and averaging (e.g., across channels, trials and/or distinct time periods) were conducted [6,7,8,10,11,12,13,77,96,97,98,99,101,102,103,104,106,107,109,110,111,115,116,119,120].

In 33 studies, the mean (average) values of the measures of cortical activity (e.g., oxyHb, deoxyHb, or TOI) were calculated over a distinct time period and were used for further statistical analysis [6,7,8,10,11,12,13,77,78,79,96,97,98,99,100,101,102,103,105,106,107,108,109,110,111,112,113,114,115,116,117,119,120]. In other studies, the median value of the proxies of cortical activity over a distinct time period [118] or the peak value obtained during the task period [104] were used to perform the statistical analysis. The fNIRS data of the entire task period were used for averaging and statistical analysis in 18 studies [11,79,97,98,99,100,101,102,103,105,107,108,113,116,117,118,119,120]. As outlined in the following, 17 studies used different time periods for the subsequent statistical analysis: 4–11 s after trial onset [6,8,106,109], 6–9 s after trial onset [12,77], 6–10 s after trial onset [114], 6–8 s after trial onset for oxyHb, and 7–9 s after trial onset for deoxyHb [7], first 10 s after trial onset [115], 2 s before trial onset to 13.5 s after trial onset [111], 5 to 19.2 s after the onset of stimulation [112], first 4 s of a trial for the preparatory period and 4–12 s after trial onset for regulatory period [110], time to peak [104], a 12 s time period [96], 90 s prior onset of cognitive testing [10], last 10 s of task period for regular statistical analysis and 100-s stimulation windows for slope method analysis [13] and a 6-s delayed boxcar function convolved with a Gaussian kernel of dispersion of 6-s full-width at half-maximum for oxyHb [78].

The cortical activity was assessed in twelve studies using soley oxyHb [77,78,79,96,99,100,103,104,106,107,111,113]; in eleven studies using oxyHb and deoxyHb [6,7,8,12,13,98,102,108,109,112,114]; in nine studies using oxyHb, deoxyHb, and totHb [11,97,101,105,110,117,118,119,120], and in six studies using a tissue oxygenation/saturation index [10,105,108,115,116,120] (for overview see Figure 4d).

In the majority of reviewed studies, statistical inference analysis was conducted using parametric methods such as analysis of variance (e.g., ANOVA) [6,7,8,11,13,77,96,97,101,102,103,105,107,108,109,113,114,115,116,117,119,120] or t-test(s) [78,79,98,99,104,111]. To account for the multiple comparison problem, a Bonferroni correction was used most frequently in the studies reviewed [6,8,77,97,102,105,107,109,110,113,114,116,119].

### 3.6. Cortical Hemodynamics during Cognitive Testing in Response to Physical Activity

In the majority of the reviewed studies cortical hemodynamics were assessed during cognitive tests targeting executive functions. Thereby, in fifteen studies, a Stroop test [6,7,8,77,79,96,97,98,101,106,109,110,115,116,119], in one study a flanker test [102], a Go/No-Go test [78], and in another one, a random number generation test [13] were used. In two studies, a modified Sternberg task [99,100], or in one study, a spatial working memory task [111], were applied to assess the cortical hemodynamic responses during a short-term working memory task. One study utilized a two-back task to quantify the cortical hemodynamic responses during working memory assessment [117]. Furthermore, in the remaining studies, a verbal fluency task [104,107], a cognitive reappraisal task [110], a visual search task [108], a reaction time task [120], and a combination of Go/No-Go task with a spatial delayed response task [105] were employed to test cognitive functions while assessing cortical hemodynamics. In most studies, cognitive tests were administered after a temporal delay of 5 min [8,77,98,108,111,113,116,117] or 15 min [7,97,99,109,114,115,119] after the cessation of acute physical activities (for an overview see Figure 4e).

While the aforementioned studies assessed prefrontal activity (e.g., via oxyHb or TOI) before physical activity, eight studies observed a higher activity of the prefrontal cortex after a single bout of aerobic activities when there was at least a one-minute delay between cessation of aerobic activities and beginning of cognitive testing [97,98,99,109,116,117,119,120]. In six studies, a higher cortical activity (e.g., higher oxyHb concentration) in prefrontal areas was noticed when the cortical activity after the cessation of aerobic activities was compared to the control condition (sitting) [6,7,8,98,100,113]. Furthermore, the activation of the prefrontal cortex during completion of cognitive testing was influenced by the type of physical activity. For instance, a lower TOI was observed during cognitive testing after high-intensity resistance activities compared to the TOI obtained after moderate aerobic activities or no physical activities [115]. Cortical activity did not change significantly after (i) slow aerobic dance [77], (ii) stretching [108], or (iii) 2 min after maximal exercise test [105]. A significantly lower oxyHb concentration during cognitive testing was noticed (i) after cycling under normobaric hypoxic conditions [114], and (ii) if the cognitive test was conducted after the cessation of moderate-intensity cycling [78]. A positive neurobehavioral relationship between measures of cortical activity in prefrontal cortex (e.g., higher oxyHb concentration) and cognitive performance (e.g., faster response times) was observed in children [119], in healthy young adults [6,8,105,109], and in healthy older adults [7]. Whereas in younger adults, concentrations of oxyHb in the left dorsolateral prefrontal cortex [6,8,109] and the left frontopolar area [8] was associated with reaction times, in healthy, older adults improved reaction times after ten minutes of moderate-intensity cycling were related to the concentration of oxyHb in the right frontopolar area [7]. In addition, one study observed that right ventrolateral oxyHb concentration was enhanced in responders (participants that showed improved task performance in post-exercise cognitive testing) during low-intensity cycling in comparison to non- responders [111].

In the long-term physical exercise studies, after a four-week intervention, an increased concentration of oxyHb in the prefrontal cortex during the cognitive testing was associated with higher weight loss [96]. Furthermore, distinct cortical hemodynamic responses during executive tests were observed after training programs with different exercise characteristics [101], but a 24 weeks Tai-Chi intervention did not significantly change the oxyHb concentration during the cognitive testing [79].

In cross-sectional studies, a higher level of cardiovascular fitness [11,12,13] or higher level of habitual physical activity [104,106,107,110,118] were associated with measures of cortical activity in the prefrontal cortex during the cognitive testing (e.g., higher oxyHb concentration and faster response times). Furthermore, in young adults, the area under the fNIRS curve (during cognitive testing) in the prefrontal cortex was associated with total sleep time [104]. In children, high levels of moderate-to-vigorous physical activity were not linked to higher oxyHb levels during cognitive testing [103]. A more detailed overview about the findings of the reviewed studies is provided in Table 1.

## 4. Discussion

In the following section, we summarize and discuss the methodology and findings of the 35 studies reviewed. With regard to (i) our discussion of the obtained findings, and (ii) general considerations concerning the application and data processing in fNIRS, we derive methodological recommendations for future studies using fNIRS to investigate the influence of physical activity on cognitive performance and cortical hemodynamics (see Table 2).

### 4.1. How Should the fNIRS Optodes be Placed?

A crucial point in neuroscience is the exact localization of functionally active parts of the human brain [126]. While fNIRS does not provide anatomical information per se, a standardized placement strategy is important to ensure (i) the comparability between studies (and neuroimaging methods, e.g., fMRI), and (ii) a reproducible data assessment of the same cortical structures when conducting repeated measurements [127,128,129,130]. The gold standard for anatomical localization of fNIRS optodes is the co-registration with fMRI [29,131]. The co-registration procedure using fMRI scans may ensure high accuracy but is often not feasible because it (i) requires an fMRI scanner, (ii) is costly, (iii) is time consuming, and (iv) may not be used in special cohorts (e.g., children, individuals with metallic implants or claustrophobia) [131]. Alternatively, a digitizer can be used to register 3-D coordinates of the fNIRS channels and project their positions onto an anatomical atlas [131,132].

The most common and practical strategy is to use the EEG 10–20 (or 10–10; 10–5) system to place the optodes [36,61,131,133]. Nearly all fNIRS studies reviewed here used this approach (see Table 1). The standardized EEG positions can be used for a virtual spatial registration of fNIRS optodes [132,134,135,136,137,138]. This procedure allows the probabilistic estimation of the most likely MNI (Montreal Neurological Institute) coordinates of the corresponding fNIRS channels [132,133,136]. Furthermore, EEG positions can be used in conjunction with IBCM (International Consortium for Brain Mapping) head model to accurately place optodes [139,140]. Freely available toolboxes, such as the “Optodes Location Decider (fOLD)” [141] or “Array Designer” [142], can support the placement of the optodes according to the desired cortical region-of-interest (ROI; e.g., derived on the basis of previous fMRI studies). These approaches enhance the anatomical specificity and sensitivity of the probe arrangement [141,142]. Another software package, the “ATLAS-viewer” (downloadable for free), can be applied (i) to ensure that the optodes are placed over a predefined ROI, and (ii) to calculate a spatial sensitivity profile of the distinct probe arrangement assuring that the used probe setup is capable of measuring the cortical compartment of the ROI [143]. In order to achieve highly reproducible hemodynamic responses and to substantially reduce the commonly observed spatial reposition error [144], it can be advantageous to use a neuronavigation system. Indeed, the spatial error significantly decreased when neuronavigation was employed, for instance, in transcranial magnetic stimulation studies [145], but also in fNIRS placing optodes with a neuronavigational device showed promising results regarding applicability and accuracy [146].

Another crucial point in fNIRS is the separation distance between the source and detector because the source–detector separation influences the measurement depth [31,54,62,68,147,148,149]. Most of the studies reviewed here employed a source–detector separation of 3.0 cm (see Figure 4a). In the literature, 4.0 cm [147] or 3.0 cm [131,150] are recommended as an optimal source–detector distance for adults. For children or infants, source–detector separations below 2.0 cm are recommended [29,131]. In general, longer source–detector separations enhance the contribution of cerebral layers to the obtained hemodynamic signal with the result that with a source–detector separation of 4.0 cm (3.0 cm), the cerebral tissue contributes to 69% (55%) to the optical signal [151]. Given (i) that at longer source-detector distances the contribution of cerebral layers to the signal is larger [148,152,153,154], (ii) that “too long” source-detector separations (exceeding 4.0–5.0 cm) may degrade the signal quality because of noisier and weaker light input to the detectors [131,149], and (iii) that application of “too long channels” reduces spatial resolution (as less channels can be used) [149], we recommend a source-detector separation of 3.0 to 5.0 cm in adults to ensure (i) that the signal quality is high, and (ii) that the fNIRS signal mainly depicts cortical activity. In addition, the freely available toolbox “Phoebe” can be used, which allows to calculate an objective measure of the signal-to-noise ratio of the optical signal (based on optode–scalp coupling of the distinct optode) before data recording [155]. These measures can improve the optode–scalp coupling and can, therefore, ensure that the fNIRS data are recorded with an appropriate signal-to-noise ratio. Furthermore, we recommend the usage of long-separation and short-separation channels (also known as “short-distance channels”; see “4.4.2. How should physiological artefacts be removed?”).

### 4.2. How fNIRS Data be Recorded?

Pivotal for neuroscience experiments assessing task-evoked brain activations is the selection of an appropriate baseline condition [21,23,156]. Based on the results of the reviewed studies, baseline brain activation should be assessed in a sitting position to ensure comparability across studies because spontaneous physiological oscillations (e.g., Mayer waves with a period length of about 10 s), which could influence the fNIRS signal quality [69,157], are posture dependent [158,159]. Indeed, substantial changes in oxyHb and deoxyHb concentration [160], as well as in TOI [161], were observed with changes in body position, which, in turn, possibly limits the comparability across studies using different body positions for data acquisition (e.g., supine vs sitting). Consequently, it seems also clear that caution should be paid when findings from fNIRS (e.g., mainly obtained in sitting position) are compared to findings of fMRI (e.g., mainly obtained in supine position). 

However, regarding the duration of baseline data assessment, there are two different approaches to be found in the reviewed studies (for an overview, see Figure 4b). In one approach, a relatively short baseline with a maximum of 30 s is used [6,7,8,12,13,79,99,100,102,104,106,108,109,113,117,120]. Other studies employ relatively long baseline measurements with more than 1 min duration [11,101,103,118]. For the choice of baseline duration, it is crucial to keep in mind that fNIRS is sensitive to mind wandering [162]. Mind wandering occurs in approximately half of the wakening hours [163], especially during situations with low perceptual requirements [164]. Hence, during the baseline period, which constitutes a situation with low perceptual requirements (e.g., still sitting), it is likely that mind wandering will occur. The wandering of the mind leads to the processing of task-unrelated thoughts [165,166] and induces stronger activations in the so-called default network [167]. Activation of the default network changes the recruitment of the prefrontal cortex [162,168] and may influence further analytic steps [36]. To prevent mind wandering, Holtzer and colleagues [169,170] incorporated a simple counting task during the baseline period. This approach could eventually minimize the potentially disadvantageous effects of mind wandering on the analysis of brain activation. However, before such a simple counting task can be recommended, its influence on brain activity and the reproducibility, have to be examined [36].

In addition to mind wandering, it should also be considered that relatively short baseline durations (e.g., 2 s) are assumed to be more affect by random physiological fluctuations and, as consequence, previous reviews recommended a baseline duration of ≈10–30 seconds to ensure appropriate signal-to-noise ratio [68]. Furthermore, especially in studies utilizing block-designs, it seems preferable to use baselines (and inter-stimulus durations) with approximately the same duration as the stimulus because (i) the refraction time (time period with reduced responsiveness) is almost as long as the stimulation phase [171], and (ii) a certain time is required to let the stimulus-evoked cortical hemodynamic responses return to the baseline level [149]. In contrast, in event-related designs, shorter time periods are used for the analysis of the baseline (e.g., 2 s before trial onset) than for the analysis of the cortical hemodynamic responses (e.g., 4–8 s after trial onset) [149,172,173,174]. Furthermore, since age affects neurovascular coupling and, in turn, the time-shape of the cortical hemodynamic response and its return to baseline levels [149,175], age should be considered as a moderating factor regarding optimal baseline duration. Moreover, it should be considered that the baseline periods between the tasks should not be a multiplier of the Mayer-wave (e.g., *n* × 0.1 Hz). Consequently, it is more appropriate to use, for instance, 15 s than 10 s for a baseline period. In addition, the duration of the baseline between trials should vary in their length (e.g., 12–18 s instead of a consistent 12 s) to diminish possible resonance effects.

However, so far, only the required minimum duration for the assessment of connectivity measures in fNIRS studies was investigated [176,177], while, to our knowledge, no study has investigated the optimal duration for baseline brain activity in fNIRS assessment yet. Hence, further investigations are needed to define optimal characteristics (e.g., duration) for baseline brain activity assessment in fNIRS [21,36]. Based on the currently available knowledge, the appropriate baseline duration should be chosen carefully and influencing factors, such as (i) mind wandering, (ii) random physiological fluctuations, (iii) study design (block design versus event-related design), and (iv) age of participants, should be taken into account [149].

In the reviewed studies, both block and event-related designs were commonly applied. The block design provides, for instance, the advantage of an adequate signal-to-noise ratio [178,179,180]; the obtained results are robust [181] and statistical power is high [179,182]. Disadvantages of the block design are (i) the impossibility of performing a trial-to-trial analysis as in event-related designs [181,183], and (ii) the occurrence of cancelling effects [184]. However, a detailed discussion of the advantages and disadvantages of different study designs in neuroimaging is beyond the scope of this article and the interested reader may find valuable information in the referenced literature [181,183,185].

### 4.3. How Should the “Optimal” Value for the DPF be Found?

DPF is a dimensionless correction factor that accounts for the increase in the optical pathlength caused by the scattering of light in biological tissue and is multiplied by the source–detector separation to estimate the “true” path length which light has travelled [23,52,121,122]. In the modified Beer–Lambert law, the DPF is used to calculate chromophore concentrations (e.g., oxyHb and deoxyHb; see Figure 2) [186,187]. If the DPF is calculated inaccurately, serious cross-talk error could occur in the determined fNIRS parameters [188], which, in turn, alter the findings and negatively affects the conclusion drawn. Given that the DPF is an such important factor, it is obviously that he should be precisely determined [47,122,123,188,189,190,191,192].

The usage of constant DPF values seems less-than-ideal because DPF values vary largely across individuals [122,123,193,194] and depend on (i) the wavelength used [122,123,192,194], (ii) the cortical area measured [122,123,190,192,195,196], (iii) the participants’ age [122,123,192,197], (iv) the size of the detector area [189], and (v) the source–detector separation [189]. Furthermore, as recently observed, the DPF can also change during the experiment [198]. Hence, we recommend the use of formulas allowing the calculation of individual, age-specific (and wavelength-specific) DPF values [122,123] or the direct quantification of the DPF value using frequency- or time-domain fNIRS (optimal solution) [36]. Arbitrary units and saturation or tissue oxygenation indexes, which has also been used in the reviewed studies, provide the advantage that they do not rely on specific DPF values [124,125].

### 4.4. How Should the Artefacts from the fNIRS Data be Removed?

In the fNIRS signal, three main sources of noise are present: (i) instrumental noise (e.g., low frequency drifts and short noise produced by light instabilities of light sources), (ii) motion-related artefacts (e.g., baseline shifts evoked by movements), and (iii) physiological oscillations (e.g., due heart beats—0.5 to 2.0 Hz, Mayer waves—0.07 to 0.13 Hz; and respiration—0.2 to 0.4 Hz) [47,69,199,200,201,202,203]. To remove those artefacts and physiological components, low-pass filters (e.g., to remove heart rate artefacts) and high-pass filters (e.g., to remove instrumental noise) are employed [29,36,47,69,204]. In most of the reviewed studies, band-pass filters (consisting of low- and high-pass filters) with a cut-off frequency of 0.7 Hz or 0.3 Hz for the low-pass filter and 0.04 Hz for the high-pass filter were applied (Figure 4c and Table S1 in Supplementary Material). Recent reviews recommend cut-off frequencies in the range of 0.5 Hz for low-pass filters and 0.01 Hz for high-pass filters [36,56,205,206]. However, the selection of appropriate filter frequencies in functional neuroimaging also depends on the stimulus protocol [207,208]. Hence, we recommend choosing the cut-off frequencies for (band-pass) filtering with care in order to avoid the unintended removal of task-evoked cortical hemodynamic responses [204].

As an alternative to the FIR/IIR bandpass filter, we recommend the use of the Savitzky-Golay filter [209], which is widely applied in fNIRS studies [210,211,212] and ensures that mostly non-related components of the evoked hemodynamic response could be removed, whereas task-related components are preserved [213]. Furthermore, the data obtained from resting-state functional connectivity could also be used to substantially reduce trial-to-trial variability (e.g., arising from low-frequency spontaneous fluctuations) in fNIRS studies [214]. In addition, it is advisable to use more sophisticated filter methods to remove physiological and motion-related noise [36,204,205,206], which cannot be removed by simple band-pass-filtering (e.g., respiration [204,215]). Examples of such advanced filter methods for the removal of motion-related and physiological noise are given in the next sections.

Additionally, open-source toolboxes such as “HOMER” [204], “NIRS Brain AnalyzIR” [216], “POTATo” [217], “FC-FNIRS” [218], “NIRS-SPM” [219] or “NIRS Analysis Package” [220], “NeuroDOT” [221], or “NIRSTORM” [222] could be used to process and analyze fNIRS data.

#### 4.4.1. How Should Motion-Related Artefacts be Removed?

To remove motion-related artefacts in fNIRS data, a collection of methods is available [199] including task-related component analysis [223,224,225], correlation-based signal improvement [226], autoregressive algorithm based filters [227], Kalman filter [228], Wiener filter [229], wavelet based filters [201,230,231,232,233], accelerometer-based filter methods [234], principal component analysis [201,235,236], Temporal Derivative Distribution Repair method [237], and/or methods based on signal reconstruction using an artificial neural network [238].

Interestingly, sophisticated filter methods like principal component analysis (PCA) were only used in one study so far [11], leaving the potential to optimize data quality with these filter methods in future studies. Wavelet filters or spline interpolation seem especially favorable to remove motion artefacts (e.g., produced by speaking during the cognitive tests) [36,205,206], whereas sudden shifts in fNIRS data (baseline shifts) could be removed using the approach developed by Scholkmann et al. [202]. Remarkably, hybrid filter techniques (e.g., combining spline interpolation method and Savitzky–Golay filtering) provide reasonable improvements in motion artefact removal (e.g., compared to existing approaches such as wavelet filters) [239]. Hence, the application of high-performing filter methods (e.g., hybrid filter methods) should be considered in future studies [239]. Furthermore, movement artefacts can also be reduced by applying multi-distance configurations of the NIRS channels, resulting in a more stable acquisition of the signals [240].

#### 4.4.2. How Should Physiological Artefacts be Removed?

Since a vast amount of literature shows that fNIRS is vulnerable to physiological noise, such as blood flow changes in the extracerebral compartment [30,70,210,211,241,242,243,244,245,246,247,248,249,250,251,252], which may cause false positive results [69], these artefacts should be reduced. A powerful tool to reduce extracerebral physiological noise is to use a combination of NIRS light channels with a short source–detector separation and with a long source–detector separation [61,152,210,211,253,254,255,256,257,258,259,260]. The integration of short-separation channels is suggested based on the following facts. The penetration depth of light is almost half the source–detector distance [148] so that channels with short separations (around 1.0 cm) mostly detect signals from non-cerebral layers [61,68,243,256,261] (see Figure 1a). The signals from these extracerebral layers can then be used to filter the signals of the “long-separation channels” (e.g., 3.0 cm source–detector separation; see Figure 1b). The optimal separation for the short-separation channels may vary across different cortical regions [253,256], but it is generally accepted that short-separation channels (i) should have a source–detector separation of < 1.0 cm [256], and (ii) should not be located further away than 1.5 cm from the standard fNIRS channel [253]. While both short-separation channels and long-separation channels were measured only in four reviewed studies [105,113,116,120], the application of additional short-separation channels should be the standard procedure in future studies [61]. However, it should also be noted that short-separation channels could be more prone to motion artefacts and that the use of “too noisy” short-separation channels as regressors could introduce additional error in the data analysis [262].

In addition, we recommend the usage of a heart rate monitor. Assessment of the heart rate can be helpful for the interpretation of the cortical hemodynamic changes measured with fNIRS since (i) the heart rate is associated with systematic changes in blood flow (blood pressure) [69], (ii) the heart rate variability provides additional information about the state of the autonomic nervous system [263,264], (iii) the heart rate variability is associated with cognitive performance and mental workload [265,266,267], and (iv) the heart rate is suggested to be a potential marker for the optimal timing of post-exercise cognitive test administration [268]. Furthermore, devices measuring electrodermal activity, respiration, or mean arterial pressure may be useful tool for the assessment of systemic changes in bodily functions that could alter the fNIRS signal [30,69,71,72,249,269,270]. Mean arterial pressure is important in order to identify the real source of the observed oxygenation changes over the head and to avoid false positive results (for a review, please see Reference [69]), and future studies should measure fNIRS signals in parallel with multiple physiological parameters [72,270,271,272]. The combination of fNIRS neuroimaging with the parallel measurement and analysis of systemic physiological signals has been termed “systemic physiological augmented fNIRS” (SPA-FNIRS) recently [72,271].

### 4.5. How Should the fNIRS Data be Processed after Filtering?

As almost all studies reviewed in this work did so, and based on another methodological fNIRS review [36], we recommend performing a baseline correction/normalization and averaging across channels and/or trials after filtering the data. Baseline correction/normalization accounts for the individual variability of fNIRS data [118,273], while averaging across channels and/or trials enhances the reproducibility of fNIRS measurements [130,274,275,276]. However, we strongly recommend reporting on which criteria the averaging procedures are based (e.g., selected channels belong to the same ROI).

Furthermore, the majority of reviewed studies used mean values calculated over a distinct time period to analyze cortical activity. The usage of mean values is preferable compared to the use of peak values because peak values are more dependant on the accurate removal of motion and other artefacts [277]. In one study the median values over the whole task period were used for statistical analysis [118]. The median values calculated across a distinct task period are less influenced by outliers as compared to mean or peak values [278]. As frequently shown [72,210,211,212,271], median values are best suited in fNIRS studies with small sample sizes that are otherwise prone to statistical effects driven by outliers [278]. However, Khan and colleagues [279] propose that several measurement parameters should be provided (e.g., mean signal value, signal peak, and the sum of peaks) in order to best describe the brain state [279]. In addition, future studies may use the variability of brain signals (e.g., oxyHb or deoxyHb) to study the effect of exercise on cognitive performance because the investigation of variability may allow a deeper understanding of the functioning of the central nervous system [280,281,282,283,284,285].

Regarding the temporal window for the analysis, it should be considered that there is in general a certain delay (e.g., ≈6 s) after stimulus representation and the peak of the cortical hemodynamic [34,41,59,131,286,287,288], whereby this latency is influenced by the performed task [289,290], and that the temporal courses of deoxyHb and oxyHb concentration changes are different [290,291,292,293,294,295]. The cortical hemodynamic response does not normally go back to the baseline level before ≈10 s (≈16 s) after stimulus presentation [296,297]. However, a consensus about an optimal temporal window for analysis has not been achieved yet [131] because what temporal duration is suited best depends on the used paradigm and the participants’ characteristics (e.g., age).

Regarding the analysis of fNIRS data, there is an ongoing debate regarding which measure (e.g., oxyHb, deoxyHb, totHB) is the optimal proxy of neuronal activation in the cortex [29,58,147,298,299]. We recommend assessing and reporting the changes of at least oxyHb and deoxyHb because (i) typically neuronal activity is assumed to be mirrored by an increase of oxyHb and a decrease in deoxyHb [34,48,49,58]; (ii) in deoxyHb signals, less physiological noise is present [58,70,144,298,300,301,302], but oxyHb signals have a higher signal-to-noise ratio as compared to deoxyHb signals [298,303]; (iii) the decrease in deoxyHb [304,305,306,307] and the increase in oxyHb [303,308] are both related to an increase in the BOLD contrast obtained in fMRI; (iv) oxyHb exhibits an acceptable high reproducibility [127,128,275,309], but deoxyHb is spatially more focused [144,310,311]; (v) deoxyHb sometimes shows an arbitrary and paradoxical signal changes [196,312,313,314,315], whereas oxyHb is assumed to be the more sensitive marker of regional blood flow changes [195,316]; (vi) pathologies may influence neurovascular coupling so that an decrease in deoxyHb does not necessarily reflect an increase in neural activity [48]; and (vii) single measures (oxyHb or deoxyHb) may not be sufficient to characterize the neurovascular response of neuronal tissue [293]. Noteworthy, sometimes researchers are confronted with atypical changes in oxyHb and deoxyHb concentration (e.g., decrease in oxyHb and increase in deoxyHb). There are several explanations for this phenomenon. Atypical changes in fNIRS signals can be caused in part by systemic physiological noise [69,71,249], by partial volume effects (e.g., caused by the mixing of signals from different tissue types) [303,306,317,318], or by the presence of certain pathophysiological changes (e.g., where the inverse response is perhaps a sign of brain activation) [48]. Furthermore, such inverse hemodynamic responses (e.g., decrease oxyHb and increase deoxyHb) could also be related to subject-specific factors (e.g., individual cognitive processes) [318]. However, as of today, this phenomenon is only partially understood, and an in-depth discussion of current explanative approaches can be found in Holper et al. [318].

While the optimal way to statistically analyze fNIRS data is still discussed and no standardized procedure has been established yet [36,69,319,320], the majority of studies reviewed used ANOVAs to statistically analyze their fNIRS data. If an ANOVA is used for statistical analysis of fNIRS data, setting ROIs as a factor should be avoided because the optical properties vary systematically across different ROIs, which could cause systematic biases in the statistical analysis of the data [6,7,8,109,114]. Furthermore, most studies reviewed used a Bonferroni correction to account for the multiple comparison problem. Notably, Singh and Dan [321] recommend the use of the false discovery rate (FDR) instead of the Bonferroni correction since FDR is less conservative than a Bonferroni correction [322,323]. Hence, future studies using fNIRS to investigate the exercise–cognition interaction should consider the application of FDR instead of a Bonferroni correction. Moreover, some authors favor the use of general linear models (GLM) to analyze fNIRS data statistically [219,319,320]. A GLM offers the possibility (i) to take the temporal shape of the hemodynamic response into account [319], and (ii) to incorporate multiple regressors (e.g., confounding signals such as scalp blood flow or heart rate) into a single statistical framework [69,320]. The latter point is especially interesting in (statistical) analysis of fNIRS data since fNIRS signals can be affected by a variety of artefacts (e.g., motion artefacts or systemic physiological artefacts) influencing the analysis and results negatively. For instance, if fNIRS data are preprocessed inappropriately (e.g., inappropriate filtering), so that the statistical assumption is violated, this will increase the type-I error substantially [262,320]. Consequently, an approach to achieve more trustworthy results is the use of sophisticated filter methods (e.g., describe in Section 4.4, Section 4.4.1, and Section 4.4.2), which appropriately remove artefacts so that they will no longer violate the assumptions of the statistical model [320]. A second approach is the application of statistical model correction methods (e.g., adopting the GLM by using “noise prewhitening”) to ensure that artefacts do not violate any statistical assumption of the used model, which, in turn, helps to obtain more reliable results [320]. From another point of view, multiple and different experimental conditions (crossed) and/or multiple measurements per experimental condition (nested) are regularly used to study the human brain [324,325]. Moreover, researchers are faced with (i) unbalanced and/or incomplete data sets, and (ii) categorical or continuous confounding variables (e.g., gender, educational level, responsiveness, genetic background), which have to be considered in the statistical analysis [324,326,327]. Hence, further fNIRS studies should also consider the application of sophisticated statistical methods such as linear mixed-effect models to account for the mentioned issues [324,325].

To sum up, in general, the statistical methods used should depend on the research question(s) and the experimental design [36,328]. For instance, whereas in event-related designs, the GLM is an appropriate method [328], simple statistics (e.g. t-tests) are commonly used in block-design studies [36,319]. Finally, the statistical methods and procedures applied to analyze fNIRS data should be chosen carefully and should consider, for instance, the experimental design, data recording and processing characteristics as well as the distribution of the recorded data (e.g., normal versus non-normal distributed data) [36]. Since a complete discussion of statistical analyses is beyond the scope of this review, the interested reader will find valuable and more detailed information in the referenced literature [219,319,320,329].

### 4.6. Cortical Hemodynamics during Cognitive Testing in Response to Physical Activity

In general, a higher activity of cortical structures (during cognitive testing) was observed after the cessation of an acute bout of physical activity (e.g., aerobic activities such as cycling) when compared to the cortical activity (i) measured before being physically active, or (ii) in a control condition (e.g., sitting). Since fNIRS signals are substantially affected by systemic physiological artefacts [70,71,72,241,242,247,248,249,251,252,271], it could be assumed that effects of physical activity on measured cortical oxygenation levels (after being physically active) are mainly caused by the systemic physiological artefacts (e.g., higher heart rate or superficial blood flow). Indeed, the findings of a methodological study suggest that fNIRS signals after the cessation of ten minutes of cycling are influenced up to approximately eight minutes by systemic physiological artefacts (depending on the intensity of the physical activity) [87]. Hence, the results of studies performing cognitive testing in close succession to physical activity (<~8 min) [8,77,78,97,98,105,108,111,113,116,117,119,120] should be treated with caution because the observed fNIRS signal changes could be, at least partly, influenced by systemic physiological artefacts. However, based on the following findings, it also becomes evident that changes in neuronal activity contributed to the measured fNIRS signal, too. For example, one study tested cognitive functions after the cessation of cycling and noticed a significantly lower cortical activity in the prefrontal cortex as compared to cortical activity before cycling [78]. Such decreased cortical activity after the cessation of moderate-intensity cycling stands in contrast to the to-be-expected effects of systemic physiological artefacts occurring after being physically active (e.g., higher heart rate). The latter would presumably induce a higher (but “false positive”) cortical activity. Hence, it seems reasonable to assume that at least a certain degree of the observed fNIRS signal is of neuronal origin if during a cognitive test, which was performed after the cessation of moderate-intensity cycling, a lower cortical activity is noticed [78]. Furthermore, if the observed higher cortical activity after an acute bout of physical activity was mainly caused by systemic physiological artefacts, the whole prefrontal cortex should be affected by the systemic physiological changes. Notably, higher cortical activity was observed only in distinct parts rather than in the whole prefrontal cortex [6,7,8,109,114] supporting the notion that the fNIRS signal is at least partly of neuronal origin. This assumption is further supported by the observation of a positive neurobehavioral relationship between cortical activity in distinct parts of prefrontal cortex and cognitive performance [6,7,8,105,109,119]. In addition to systemic physiological changes, it could be speculated that the commonly observed increase in cortical activity after being physically active is attributable to learning effects, which may occur in a repeated-measures design. However, a significantly higher cortical activity during cognitive testing was even observed in studies employing a counterbalanced order of conditions (e.g., cycling versus sitting) [6,7,8]. Hence, it is unlikely that the pronounced cortical activation seen after physical activity is predominantly caused by learning effects. This assumption is underpinned by findings of decreased cortical activity in response to learning (e.g., motor learning) [330,331,332].

The observations that (i) oxyHb concentration did not increase significantly after slow dancing [77], stretching [108], or after maximal exercise testing [105] is likely to be related to the moderating effects of (i) the characteristics of the physical activities (e.g., to low intensity (dancing, stretching)), and (ii) the study methodology (e.g., time point of cognitive test administration; i.e., 2 min after maximal exercise test), which are known to influence cognitive performance [17,333,334,335]. The lower concentration of oxyHb in DLPFC after cycling under normobaric hypoxic conditions [114] may explain why cognitive performance is commonly found to be lower after exposure to hypoxia [336].

Regarding long-term exercise studies and cross-sectional studies, the link between a higher level of cardiorespiratory fitness and/or physical activity and higher levels of cortical activity [10,11,12,13,96,101,104,106,107,112,118] is in accordance with the cardiorespiratory fitness hypothesis, which claims that cardiorespiratory fitness has a positive influence on cerebrovascular structure and function [90,337,338,339]. However, in none of these longitudinal and cross-sectional studies (e.g., using continuous-wave NIRS), physiological artefacts were corrected by measures of systemic physiological changes (e.g., extracerebral noise via short-separation channel regression). While the relationships between measures of cortical activity and cognitive performance [10,12,104,118] suggest that the fNIRS signals stem to a certain degree from neuronal activity, the application of, for instance, short-separation channel regression, allowing for a more accurate localization of the signal origin (extracerebral changes versus neuronal activity changes). As a consequence of the improved signal quality (e.g. through short-separation channel regression), the conclusions derived from fNIRS-measured proxies of cortical activity (e.g., oxyHb and deoxyHb) become more valid and reliable, which, in turn, fosters our understanding of the relationship of physical activity, cortical hemodynamics and cognition.

To sum up, based on the evidence that (i) systematic artefacts may contaminate fNIRS signals up to 8 min after being physically active [87], and (ii) higher effect sizes were evident after a temporal delay compared to cognitive testing immediately after being physically active [333], we recommend that future studies aimed at investigating the effects of an acute bout of physical activities incorporate a temporal delay (e.g., ≈8 min) between the cessation of the physical activity and the beginning of cognitive testing. Furthermore, we recommend the assessment of multiple physiological measures (see Section 4.4.2 “How Should Physiological Artefacts be Removed?”) to improve the signal quality and, in turn, validity of the observations. 

Additionally, in future studies, follow-up measurements should be undertaken because only four of the reviewed studies performed follow-up testing [97,98,111,119], which limits our knowledge about the temporal course of the relationship between physical activity, cortical hemodynamics, and cognition. Finally, the following general recommendations should also be considered when designing studies investigating the influence of physical activity on cognition while measuring cortical hemodynamics with fNIRS:

(i) Chronobiological effects (circadian variability) affects cognitive performance [340,341,342], although it is reported that the hemodynamic response is relatively unaffected by circadian variability [343].
(ii) Cognitive tasks that necessitate (inner) speech could induce hypocapnia (i.e. a decrease in the arterial carbon dioxide (CO_2_) concentration in the blood), which provokes a cerebral vasoconstriction and lower cerebral blood flow that results in a reduced concentration of total hemoglobin and thus also oxygenated and deoxygenated hemoglobin [270,344,345,346]. Exemplarily, if the task is changing the respiration (rate or depth) of the subject, the fNIRS data will likely be influenced by this CO_2_ effect and will not represent changes in neurovascular coupling primarily.
(iii) Participants should be familiarized with the cognitive test to avoid (or at least minimize) learning effects [347,348] and to increase the reproducibility of the observed cognitive effects [349].
(iv) The biological sex of the participants influences the relationship between physical activity and cognition [350,351,352,353]. Sex-specific changes are also noticed in fNIRS signals obtained during cognitive testing [354,355]. Hence, the biological sex of the participants should be considered as a moderating factor in future studies.

## 5. Conclusions

All in all, the application of neuroimaging tools (e.g., fNIRS) is pivotal to better understand the influence of physical-activity-induced mechanisms on cognitive performance. Based on the advantages of fNIRS, this neuroimaging method is a promising tool to shed light on physical-activity-induced functional brain changes (e.g., changes in cortical hemodynamics during cognitive testing). However, currently no standardized procedures with respect to the application of fNIRS and processing of fNIRS data in exercise–cognition science have been established which clearly limits the comparability across studies. To come closer to more standardized protocols, this systematic review aims to summarize the methodological details of studies applying fNIRS to investigate the influence of physical activity on cognitive performance and underlying neurobiological processes (e.g., cortical hemodynamics). Therefore, 35 fNIRS studies were carefully reviewed and based on our finding’s, methodological recommendations for further fNIRS studies in the field of exercise–cognition were derived (see Table 2). Hopefully, this methodology-focused, systematic review encourages further research in this field which is strongly needed to better understand underlying neurobiological mechanisms of exercise–cognition interaction. A growing knowledge in exercise–cognition interaction may contribute to the development of more efficient physical intervention approaches [356] aiming to prevent (or deaccelerate the onset of) age-related cognitive decline which is associated with neurological diseases such as dementia [357,358,359].

## Figures and Tables

**Table 1:** Overview about the population characteristics, fNIRS methodology and data processing, exercise characteristics and cognitive testing, and main outcomes of reviewed studies.

First Author	Sample Characteristics—Number of Participants (*n*)/Mean Age in Years ± SD	Main Findings	Region of Interest (ROI)
**Studies conducting an acute bout of physical activity**			
**Ando et al. [120]**	Healthy young adults *n* = 10 m/25.1 ± 3.4	After cycling vs. prior cycling (normoxia): - ↑ oxyHb and TOI in rt. PFC during CT	rt. PFC
**Bediz et al. [117]**	Healthy young adults HP *n* = 18 m/21.0 ± 2.6 LP *n* = 17 m/20.6 ± 2.1	After cycling vs. prior cycling: - ↑ oxyHb and total Hb in md. PFC during CT in both groups - ↑ deoxyHb in md. PFC during CT in HP - ↑ oxyHb and totHb in lt. and md. PFC during CT in HP - PP is correlated with oxyHb	lt., rt. and md. PFC
**Byun et al. [8]**	Healthy young adults *n* = 25 (12 f, 13 m)/20.6 ± 1.0	After cycling vs. control condition (sitting): - ↑ oxyHb in lt. DLPFC and lt. FPA during CT - oxyHb in lt. DLPFC and lt. FPA are associated with RT in CT	lt. and rt. DLPFC, VLPFC; FPA
**Chang et al. [115]**	Healthy young adults HC *n* = 9 f/21.8 ± 1.4 HIR *n* = 9 f/21.1 ± 1.6 MIC *n* = 9 f/20.4 ± 1.5 HIA *n* = 9 f/22.1 ± 1.4	Post-test (neutral condition): - ↓ TOI in lt. PFC (HIR vs. CON/MIC) Post-test (incongruent condition): - ↓ TOI in lt. PFC (HIR vs. CON/MIC) TOI in rt. PFC (HIR vs. CON/MIC/HIA)	lt. and rt. PFC
**Endo et al. [98]**	Healthy young adults *n* = 13 (8 f, 5 m)/23.0 ± 1.0	After cycling vs. prior cycling: - ↑ oxyHb in DLPFC during CT (40% and 60% intensity) After cycling vs. control condition (sitting): - ↑ oxyHb in DLPFC during CT (60% intensity) (results for 15 min exercise condition/test administration 5 min after exercise cessation)	lt. and/or rt. DLPFC
**Faulkner et al. [116]**	Healthy young adults *n* = 17 m/24.6 ± 4.3	After cycling vs. prior cylcing: - ↑ *r*SO_2_ in PFC during CT	lt. and rt. PFC
**Faulkner et al. [97]**	Patients with TIA and HC TIA *n* = 11 (2 f, 9 m)/65.0 ± 10.0 HC *n* = 15 (2 f, 13 m)/62.0 ± 7.0	After cycling vs. prior cycling: - ↑ oxyHb, deoxyHb and totHb in PFC during CT (for test administration 1.5 min after exercise cessation)	dominant side of PFC ^1^
**Hyodo et al. [7]**	Healthy older adults *n* = 16 (5 f, 28 m)/69.3 ± 3.5	After cycling vs. control condition (sitting): - ↑ oxyHb in rt. FPA during CT - oxyHb in rt. FPA is associated with RT in CT	lt. and rt. DLPFC, VLPFC; FPA
**Hyodo et al. [77]**	Healthy older adults *n* = 13 (6 f, 7 m)/69.7 ± 2.7 (f); 69.3 ± 2.8 (m)	Cycling vs. dancing: - no significant differences between timepoints or groups	lt. and rt. DLPFC, VLPFC; FPA
**Kujach et al. [109]**	Healthy, sedentary young adults *n* = 25 (9 f, 16 m)/20.7 ± 1.9 (f); 21.1 ± 1.9 (m)	After cycling vs. prior cycling: - ↑ oxyHb in lt. DLPFC post-exercise during CT - oxyHb in lt. DLPFC is associated with RT in CT	lt. and rt. DLPFC, VLPFC; FPA
**Lambrick et al. [119]**	Healthy children *n* = 20 (11 f, 9 m)/8.8 ± 0.8	After running vs. prior running: - ↑ oxyHb and totHb in PFC post-exercise during CT (at all three time points) - ↑ oxyHb and totHb in PFC post-exercise during CT (1 min vs. 15 min and 30 min post-exercise) - total Hb is associated with Stroop completion time (for intermittent group)	dominant side of PFC ^1^
**Moriya et al. [99]**	Patients suffering from stroke *n* = 11 (4 f, 7 m)/69.6 ± 12.0	After cycling vs. prior cycling: - ↑ oxyHb in rt. PFC post-exercise during CT	rt. and lt. PFC
**Murata et al. [78]**	Healthy young adults *n* = 15 (6 f, 9 m)/21.7 ± 2.4; 21.6 ± 3.0 (f); 21.8 ± 2.2 (m)	After cycling vs. prior cycling: - ↓ lt. DLPFC and SMA post-exercise during CT (Go-trials)	rt. and lt. DLPFC, SMA
**Ochi et al. [114]**	Healthy young adults *n* = 15 (8 f, 7 m)/20.7 ± 2.1 (18-25)	After cycling (normobaric hypoxia) vs. control condition (sitting/normobaric hypoxia): - ↓ oxyHb in lt. DLPFC post-exercise during CT - oxyHb in lt. DLPFC is associated with RT in CT	lt. and rt. DLPFC, VLPFC; FPA
**Sudo et al. [108]**	Healthy young adults Stretching group *n* = 8 m/23.9 ± 2.3 Control group *n* = 8 m/23.8 ± 2.1	After stretching vs. prior stretching: - oxyHb, deoxyHb and TOI in lt. PFC no significant differences between timepoints or groups	lt. PFC
**Sudo et al. [105]**	Healthy young adults Cycling group *n* = 18 m/23.2 ± 2.1 Control group *n* = 14 m/22.3 ± 2.3	After cycling vs. prior cycling: - oxyHb, deoxyHb, totHb and cerebral oxygenation in rt. PFC no differences during CT - Δcerebral oxygenation (TOI) is associated with Δ reaction time	rt. PFC
**Tsuchiya et al. [113]**	Healthy young adults *n* = 25 (19 f, 6 m)/19.88 ± 0.60 (18-21)	Housework activities vs. control condition: - ↑ oxyHb (trend) in rt. VLPFC during CT (Stroop interference score between post- and pre-sessions)	lt. and rt. DLPFC, VLPFC; FPA
**Tsujii et al. [100]**	Healthy older adults *n* = 14 (9 f, 7 m)/65.9 ± 1.0	After cycling vs. control condition (sitting): - ↑ oxyHb in lt. PFC during CT	rt. and lt. PFC
**Yamazaki et al. [111]**	Healthy young adults *n* = 14 (6 f, 8 m)/22 ± 0.6	After recumbent cycling vs. prior cycling: - oxyHb no difference in the ROI’s during CT Responders vs. Non-Responders ^2^: - ↑ (maximum peak) oxyHb in rt. VLPFC during exercise	lt. and rt. DLPFC, VLPFC; FPA
**Yanagisawa et al. [6]**	Healthy young adults *n* = 20 (3 f, 17 m)/21.5 ± 4.8	After cycling vs. control condition (sitting): - ↑ oxyHb in lt. DLPFC post-exercise during CT - oxyHb in lt. DLPFC is associated with RT in CT	lt. and rt. DLPFC, VLPFC; FPA
**Studies conducting long-term physical exercises**			
**Chen et al. [102]**	Healthy young adults *n* = 42 (26 f, 16 m)/22.5 ± 2.0	Post-test vs. pre-test: - ↑ oxyHb in lt. PFC in BMB (incongruent condition)	lt. and rt. PFC
**Coetsee et al. [101]**	Healthy older adults HIIT *n* = 13 (10 f, 3 m)/64.5 ± 6.3 MCT *n* = 13 (10 f, 3m)/61.6 ± 5.8 ReT *n* = 22 (15 f, 7 m)/62.4 ± 5.1 CON *n* = 19 (11 f, 8 m)/62.5 ± 5.6	Post-test vs. pre-test: - ↑ oxyHb in lt. PFC in CON (naming condition) - ↑ deoxyHb in lt. PFC in MCT and HIIT (naming and executive condition) - ↓ THI in lt. PFC in MCT (naming and executive condition) - ↓ oxyHb in lt. PFC in ReT (Stroop interference effect) - ↓ THI in lt. PFC in ReT and MCT (Stroop interference effect)	lt.and rt. PFC
**Wang et al. [79]**	Healthy older adults *n* = 12 (8 f, 4 m)/64.25 ± 3.14 (60 - 68)	Post-test vs. pre-test (after Tai-Chi intervention): - no significant differences between timepoints	frontal cortex
**Xu et al. [96]**	Obese young adults *n* = 31 (12 f, 19 m)/18.2 ± 3.2	Participants with higher weight reduction vs. participants with lower weight reduction: - ↑ oxyHb in lt. and rt. DLPFC, VLPFC; FPA during CT	lt. and rt. DLPFC, VLPFC; FPA
**Cross-sectional studies**			
**Albinet et al. [13]**	Healthy older adults *n* = 40 f/60-77 (low-fit group *n* = 17/high-fit group *n* = 17)	High-fit group vs. low-fit group: - ↑ oxyHb in rt. DLPFC Low-fit group: - ↑ oxyHb in rt. DLPFC compared to lt. DLPFC Correlation between hemodynamic responses during CT and physical fitness: - relationship between aerobic fitness (assessed via VO_2_ *max*) and cognitive performance is partly mediated by slope coefficient of oxyHb in the rt. DLPFC (at 1.5 s pace condition)	lt. and rt. DLPFC
**Cameron et al. [118]**	Healthy young adults *n* = 52 f/20.7 ± 2.3	Correlation between hemodynamic responses during CT and measures of physical activity or cognition: - higher chronic physical activity level is linked to higher oxyHb and superior cognitive performance - correlation between oxyHb and deoxyHb with RT (difficult condition)	rt. PFC
**Dupuy et al. [11]**	Healthy younger adults *n* = 22 f/24.6 ± 3.6 (19-34) Healthy older adults *n* = 36 f/62.9 ± 5.4 (55-72)	High-fit individuals vs. low-fit individuals: - ↑ oxyHb in rt. inferior frontal gyrus during CT (naming and executive condition) - ↑ totHb in rt. inferior frontal gyrus during CT (naming and executive condition)	lt. and rt., ant. and post. DLPFC and VLPFC
**Fabiani et al. [112]**	Healthy, high-fit older adults *n* = 20 (11 f, 9 m)/70.3 ± 4.2 Healthy, low-fit older adults *n* = 24 (13 f, 11 m)/72.2 ± 5.2	High-fit older adults vs. low-fit older adults: - VO_2_ peak is correlated with oxyHb but not deoxyHb changes	lt. and rt. occipital cortex
**Giles et al. [110]**	Healthy young adults *n* = 74 (50 f, 24 m)/19.55 ± 0.27	Correlation between hemodynamic responses during CT and habitual exercise level: - greater habitual exercise level is associated with↓ oxyHb and totHb during CT (negative and neutral pictures/during preparatory period)	ant. PFC and DLPFC
**Hyodo et al. [12]**	Healthy older adults *n* = 60 m/70.3 ± 3.2	Correlation between hemodynamic responses during CT and physical fitness or cognition: - activation in lt. DLPFC is positively associated with VT - activation in lt. DLPFC is negatively associated with Stroop interference time	lt. and rt. DLPFC
**Kato et al. [104]**	Healthy young adults *n* = 23 (10 f, 13 m)/22.0 ± 2.2	Correlation between hemodynamic responses during CT and measures of physical activity or sleep duration: - exercise amount is associated with the AUC during CT - exercise amount is correlated with reaction time on fNIRS - percentage of correct responses in CPT-IP are correlated with peak oxyHb - total sleep time is associated with the AUC during CT	lt. and rt. frontal areas
**Makizako et al. [107]**	Healthy older adults *n* = 20 (10 f, 10 m)/76.1 ± 6.7 (66-89)	Group with high physical activity level vs. group with low physical activity level: - ↑ oxyHb in lt. and rt. IFG during CT	lt. and rt. IFG
**Matsuda et al. [106]**	Healthy young adults *n* = 40 (15 f, 25 m)/20.4 ± 1.1	Group with high physical activity level vs. group with low physical activity level: - ↑ oxyHb in lt. DLPFC during CT (Interference condition)	lt. DLPFC
**Mücke et al. [103]**	Healthy children *n* = 50 (24 f, 26 m)/10.6 ± 0.3 (low MVPA *n* = 20/high MVPA *n* = 30)	Group with low MVPA vs. group with high MVPA: - no significant differences in cortical activity between group with low MVPA and group with high MVPA	lt. and rt. ant. PFC; lt. and rt. intermediate and md. frontal region
**Suhr and Chellenberg [10]**	Healthy, older adults *n* = 22 (17 f, 5 m)/68.26 ± 8.39 (54-89)	Correlation between hemodynamic response during CT and measures of physical activity or cognition: - hours of physical activity are associated with *r*SO_2_ - memory performance is correlated with *r*SO_2_	lt. and rt. DLPFC

Ant: anterior; AUC: area und the curve; BMB: Baduanjin Mind-Body Intervention; CON: control group; CPT-IP: continuous performance test-identical pairs; CT: cognitive testing; deoxyHb: deoxygenated hemoglobin; DLPFC: dorsolateral prefrontal cortex; f: female; FPA: frontopolar area; HC: healthy controls; HIA: high-intensity aerobic exercise; HIIT: high-intensity aerobic interval training; HIR; high-intensity resistance training; HP: high performer; IFG: inferior frontal gyrus; LP: low performer; lt.: left; m: male; MCT: moderate continuous aerobic training; md.: middle; MIC: moderate-intensity exercise combining resistance training and walking; min: minute; MVPA: moderate-to-vigorous physical activity; *n* = number of participants; oxyHb: oxygenated hemoglobin; PFC: prefrontal cortex; post.: posterior; PP: peak performance in exercise test; ROI: region of interest; ReT: resistance training; rt.: right; RT: reaction time; s: second; SD: standard deviation; SMA: supplementary motor area; THI: total hemoglobin index; TIA: patients with transient ischemic attack; TOI (or *r*SO_2_): tissue oxygenation index; totHb: total hemoglobin; VLPFC: ventrolateral prefrontal cortex; VO_2_ *max*/VO_2_ *peak*: maximal oxygen uptake; vs.: versus; VT: ventilatory threshold; ↑: significant increase; ↓: significant decrease / ^1^ In right-side dominant participants the probe is placed over right prefrontal cortex while in left-side dominant participants the probe is placed over left prefrontal cortex. / ^2^ Responders are participants who showed improved task performance in cognitive testing conducted at 5 min after cycling. Non-responder showed no significant improvement in cognitive functions after performing the acute bout of cycling.

**Table 2:** Recommendations for fNIRS application, fNIRS data processing and fNIRS data analysis

**fNIRS recording**	
**Optode placement**	Optimal solution: ▪ Use a neuronavigational approach Alternative solution: ▪ Use 10-20 (10-10 or 10-5) international EEG-system ➢ If MRI scan is possible → Co-registration ➢ If MRI scan is not possible → Registration via 3-D-Digitizer *or* → Virtual spatial (probabilistic) registration
**Source–detector separation**	▪ At least 3.0 cm for “long-separation channels” ▪ Around 0.8 cm for “short-separation channels”
**Baseline recording**	▪ Record baseline in sitting position ▪ Choose an appropriate baseline duration (e.g., with regard to study design) ▪ Ensure that the fNIRS channels have a good SNR (e.g., look for blood volume pulsation)
**fNIRS data processing: conversion and artefact removal**	
**Conversion of optical density changes into concentration changes of chromophores (e.g. oxyHb, deoxyHb, totHb)**	▪ Apply modified Beer–Lambert law with appropriate μa and DPF values
- DPF value determination	Optimal solution: ▪ Direct quantification of DPF values using frequency- or time-domain fNIRS Alternative solution: ▪ Use formulas allowing the calculation of individual, age-specific, and wavelength-specific DPF values
**Artefact removal**	
Removal of motion artefacts *	▪ Use of high-performing methods (e.g., Wavelet filtering or hybrid filter methods)
Removal of physiological artefacts	▪ Use of high-performing methods (e.g., SDS regression to filter out extracerebral signal components)
General artefact removal	▪ Use a band-pass filtering with appropriate cut-off frequencies (e.g. considering stimulus or task paradigm)
**fNIRS data processing: further analysis**	
**Detrending**	▪ Perform baseline correction or normalization
**Analysis**	▪ Perform averaging across channels and trials *or* perform GLM analysis ^#^ ▪ Choose an appropriate temporal window (e.g., consider delay in hemodynamic responses) ▪ Use at least oxyHb and deoxyHb for statistical analysis

deoxyHb: deoxygenated hemoglobin; DPF: differential path length factor; EEG: electroencephalography; fNIRS: functional near-infrared spectroscopy; GLM: general linear model; μa: absorption coefficient; MRI: magnetic resonance imaging; oxyHb: oxygenated hemoglobin; SDS: short-separation channel (also known as short-distance channel); SNR: signal-to-noise ratio/* Filtering of motion artefacts can also be conducted on optical density data (before conversion into concentration changes) depending on the used filter methods and/or software solution. / ^#^ Please note, if distinct types of GLM are used (e.g., GLM with model correction methods) the processing steps are divergent from those shown in the table and some of the given recommendations do not apply in this particular case.

## Supplementary Materials

The following are available online at http://www.mdpi.com/2077-0383/7/12/466/s1, Figure S1: Overview on (**a**) number of subjects investigated, (**b**) sampling frequencies (**c**) wavelengths, (**d**) number of channels, (**e**) differential pathlength factors (DPF), (**f**) types of physical activities/exercises and (**g**) durations of physical activities/exercises. Table S1: Overview about data recording (e.g., used sampling frequency, wavelengths, number of measurement channels, fNIRS devices, optode placement and source-detector separation), data processing (e.g., filter frequencies, DPF values, markers of cortical activity) and characteristics of physical activities (e.g., type, duration and intensity of the physical activity) in the studies reviewed.
