# Supplementary material for: Applications of Functional Near-Infrared Spectroscopy (fNIRS) Neuroimaging in Exercise–Cognition Science: A Systematic, Methodology-Focused Review
Source: J Clin Med. 2018 Nov 22;7(12):466. doi: 10.3390/jcm7120466 (PMC6306799; doi:10.3390/jcm7120466)
Supplement: Supplementary file 1 [file jcm-07-00466-s001.pdf]

In this supplementary material to the original (Herold et al. (2018), "*Applications of Functional Near-Infrared Spectroscopy (fNIRS) Neuroimaging in Exercise-Cognition Science: A Systematic, Methodology-Focused Review*"), we provide information about (i) the different types of near-infrared spectrometers and (ii) details of the reviewed studies regarding data recording (e.g., used sampling frequency, wavelengths, number of measurement channels, fNIRS devices, optode placement and source-detector separation), data processing (e.g., filter frequencies, DPF values, markers of cortical activity) and characteristics of physical activities (e.g., type, duration and intensity of the physical activity).

Supplementary information to types of near-infrared spectrometers

Currently four methods how to implement (functional) near-infrared spectroscopy ([f]NIRS) technically exist: (i) continuous-wave NIRS (CW-NIRS), (ii) spatially resolved NIRS (SRS-NIRS), (iii) frequency domain NIRS (FD-NIRS) and (iv) time domain NIRS (TD-NIRS).

(i) In continuous-wave NIRS devices, light with a distinct intensity is emitted into the tissue via an emitter. When the light leaves the tissue at a distinct point (see Figure 1 in the main manuscript), the intensity of outgoing light is determined with a detector. The changes of light intensity (e.g., attenuation) are used to calculate relative concentration changes of chromophores such as oxyHb and deoxyHb [1–5]. The method is able to determine absolute changes in the attenuation coefficient and the fNIRS signals obtained reflect relative concentration changes (e.g., relative to first measured values).

(ii) SRS-NIRS is a type of CW-NIRS but in SRS-NIRS the outgoing light is detected at least at two different points. Based on the information of the two detectors, local gradients of light attenuation could be calculated and used to assess absolute changes in the concentration of chromophores [6, 7].

(iii) In NIRS devices that operate in FD mode, light with a distinct intensity is continuously emitted into the tissue and the amplitude of the incident light is modulated at specific frequency in MHz range. By quantifying the phase shift (delay) and light attenuation of the detected light, the absorption and scattering properties of the tissue could be determined. The information about scattering and absorption properties of the tissue are used to calculate absolute concentration values of the chromophores [1–5].

(iv) In time domain NIRS extremely short light impulses are emitted into the tissue. Using a detector which is placed at a certain distance from the light emitting source, the arrival times, temporal distribution and shape of the temporal distribution of the photons leaving the tissue are measured. Based on the information obtained, scattering and absorption properties of the tissue can be determined which were, in turn, used to calculate the absolute concentrations of chromophores [1–5].

A more detailed overview about differences between the types of near-infrared spectroscopy devices could be found in the referenced literature [1–5, 7–10]. However, since SRS-NIRS devices, FD-NIRS and TD-NIRS offer the advantage to measure absolute concentration changes, especially the latter is more expensive and has a slower acquisition rate than CW-NIRS devices [2, 3, 11]. Furthermore, the time of flight is a noisier parameter (compared to light attenuation measurement) which makes TD-NIRS devices not useful to detect small functional activations [1, 2, 11]. An overview about the type of fNIRS devices which were used in the studies reviewed is provide in Table S1.

Supplementary table

Table S1. Overview about data recording (e.g., used sampling frequency, wavelengths, number of measurement channels, fNIRS devices, optode placement and source-detector separation), data processing (e.g., filter frequencies, DPF values, markers of cortical activity) and characteristics of physical activities (e.g., type, duration and intensity of the physical activity) in the studies reviewed.

**First Author**	**1.) Sampling frequency**<br>**2.) Wavelengths**<br>**3.) Number of channels**<br>**4.) Producer and type of fNIRS device (measurement mode)**	**1.) Optode placement**<br>**2.) Source-detector separation**<br>**3.) Baseline condition (duration)**<br>**4.) Data filtering**<br>**5.) DPF**<br>**6.) Final data processing**<br>**7.) Activation parameters / temporal window of statistical analysis**	**1.) Previous GXT (parameter)**<br>**2.) (Cardiorespiratory) fitness level**<br>**3.) Type of physical activity**<br>**4.) Duration of physical activity**<br>**5.) Intensity of physical activity**<br>**6.) Cognitive test**<br>**7.) Cognitive test administration after the cessation of the acute bout of physical activity**
**Studies conducting an acute bout of physical activity**			
**Ando et al. [12]**	1.) 10 Hz<br>2.) 780, 810, 830 nm<br>3.) 2 channel<br>4.) BOM-L1 TRW, Omegawave, Tokyo, Japan (CW; SRS)	1.) on forehead using landmarks (nasion, eyebrow & hairline)<br>2.) 2.0 cm (SDS) & 4.0 cm<br>3.) sitting (30 s before CT)<br>4.) n.a.<br>5.) age-dependent value (4.99 + 0.067 × age $^{0.814}$)<br>6.) baseline normalization; averaging; use of short-separation channel<br>7.) mean of oxyHb, deoxyHb, totHb & TOI (= oxyHb/totHb × 100) / entire task period	1.) yes ($VO_{2\,peak}$)<br>2.) 49.1 ± 5.7 $\frac{mL}{kg \cdot min}$<br>3.) cycling (normoxia / hypoxia)<br>4.) 19.5 min<br>5.) 40%, 60%, and 80% $VO_{2\,peak}$ (in successive order during the single bout; under normoxia and hypoxia)<br>6.) reaction time measurement<br>7.) 1 min
**Bediz et al. [13]**	1.) 2 Hz<br>2.) 730, 850 nm<br>3.) 16 channels<br>4.) Imager 100, fNIR Devices LLC, MD, USA (CW)	1.) on the forehead<br>2.) 2.5 cm<br>3.) sitting (20 s before CT)<br>4.) LPF at 0.1 Hz; visual inspection<br>5.) n.a.<br>6.) baseline correction, averaging<br>7.) mean of oxyHb, deoxyHb & totHb / entire task period	1.) no<br>2.) 800.7 ± 77.3 PP in Watt (HP) / 634.3 ± 89.3 PP in Watt (LP)<br>3.) cycling (Wingate Anaerobic test)<br>4.) 30 s (10 min with warm-up and cool down)<br>5.) anaerobic maximal<br>6.) working memory (2-back test)<br>7.) 5 min

**Byun et al. [14]**	1.) 10 Hz<br>2.) 785, 830 nm<br>3.) 48 channels<br>4.) ETG-7000, Hitachi Medical Corp., Tokyo, Japan (CW)	1.) EEG-system<br>2.) 3.0 cm<br>3.) sitting (0-2 s before trial onset)<br>4.) HPF at 0.04 Hz; LPF at 0.3 Hz<br>5.) n.a. (arbitrary unit)<br>6.) baseline correction; averaging<br>7.) mean of oxyHb & deoxyHb / 4–11 s after trial onset	1.) yes ($VO_{2\,peak}$)<br>2.) 38.3 ± 7.2 $\frac{mL}{kg \cdot min}$<br>3.) cycling<br>4.) 10 min<br>5.) circa 30% of subjects $VO_{2\,peak}$<br>6.) executive functions (Stroop test)<br>7.) 5 min
**Chang et al. [15]**	1.) 1 Hz<br>2.) 775, 810, 850 nm<br>3.) 2 channels<br>4.) NIRO-200, Hamamatsu Photonics K.K., Hamamatsu, Japan (CW; SRS)	1.) n.a.<br>2.) n.a.<br>3.) sitting<br>4.) n.a.<br>5.) n.a. (TOI)<br>6.) averaging<br>7.) mean of TOI / first 10 s after trial onset	1.) yes (ReT; 1 RM) / no (MIC & HIA; HRR calculated)<br>2.) n.a<br>3.) HIR, MIC (resistance and walking training), HIA (running)<br>4.) around 40 min (10 min warm-up, 30 min exercising)<br>5.) 80 % 1RM (HIR); 50-60 HRR (MIC); 80% HRR (HIA)<br>6.) executive functions (Stroop test)<br>7.) 15 min
**Endo et al. [16]**	1.) 1 Hz<br>2.) 775, 810, 850 nm<br>3.) 2 channels<br>4.) NIRO-200, Hamamatsu Photonics K.K., Hamamatsu, Japan (CW; SRS)	1.) EEG-system<br>2.) 4.0 cm<br>3.) sitting (60 s before CT)<br>4.) n.a.<br>5.) n.a.<br>6.) baseline correction; averaging<br>7.) mean oxyHb & deoxyHb/ entire task period	1.) yes (Ex_{max})<br>2.) 34 ± 2 W (20 % Ex_{max}); 68 ± 5 W (40 % Ex_{max}); 103 ± 7 W (60 % Ex_{max})<br>3.) cycling<br>4.) 15 min (1 min cool down)<br>5.) 20, 40, or 60 % of subjects Ex_{max}<br>6.) executive functions (Stroop test)<br>7.) 5 min / 20 min
**Faulkner et al. [17]**	1.) 0.25 Hz<br>2.) 730, 810 nm<br>3.) 4 channels<br>4.) 5100C INVOS, Somanetics Corporation, Troy, MI, USA (CW; SRS)	1.) on the forehead<br>2.) 3.0 & 4.0 cm<br>3.) sitting (10 min before CT)<br>4.) HPF<br>5.) n.a. (rSO_2)<br>6.) averaging; use of short-separation channel<br>7.) mean of rSO_2 / entire task period	1.) yes (GET / $VO_{2\,peak}$)<br>2.) upright = 49.1 ± 5.3 $\frac{mL}{kg \cdot min}$ / recumbent = 45.1 ± 5.4 $\frac{mL}{kg \cdot min}$<br>3.) upright and recumbent cycling<br>4.) 30 min<br>5.) 45-60% of subjects $VO_{2\,peak}$<br>6.) executive functions (Stroop test)<br>7.) 5 min
**Faulkner et al. [18]**	1.) 10 Hz<br>2.) 760, 850 nm<br>3.) 3 channels	1.) EEG-system<br>2.) 3.0, 3.5 & 4.0 cm<br>3.) sitting (10 min before CT)<br>4.) HPF	1.) yes (GET / $VO_{2\,peak}$)<br>2.) upright TIA / HC = 28.2 ± 7.7 / 39.1 ± 8.9 $\frac{mL}{kg \cdot min}$ / recumbent TIA / HC = 28.5 ± 8.6 / 34.8 ± 8.9 $\frac{mL}{kg \cdot min}$

	4.) PortaLite, Artinis, Medical Systems BV, The Netherlands (CW; SRS)	5.) constant value (4.0)<br>6.) baseline correction; averaging<br>7.) mean of oxyHb, deoxyHb & totHb / entire task period	3.) upright and recumbent cycling<br>4.) 30 min<br>5.) 45-60% of subjects $VO_{2\,peak}$<br>6.) executive functions (Stroop test)<br>7.) 1.5 min / 15 min
**Hyodo et al. [19]**	1.) 10 Hz<br>2.) 785, 830 nm<br>3.) 48 channels<br>4.) ETG-7000, Hitachi Medical Corp., Tokyo, Japan (CW)	1.) EEG-system<br>2.) 3.0 cm<br>3.) sitting (0-2 s before trial onset)<br>4.) HPF at 0.04 Hz; LPF at 0.7 Hz<br>5.) n.a. (arbitrary unit)<br>6.) baseline correction; averaging<br>7.) mean of oxyHb & deoxyHb / 6-8 s after trial onset for oxyHb and 7-9 s after trial onset for deoxyHb	1.) yes (VT)<br>2.) 14.4 ± 3.0 $\frac{mL}{kg \cdot min}$<br>3.) cycling<br>4.) 10 min<br>5.) VT / corresponds to 40% to 59% of subjects $VO_{2\,peak}$<br>6.) executive functions (Stroop test)<br>7.) 15 min
**Hyodo et al. [20]**	1.) 10 Hz<br>2.) 780, 805, 830 nm<br>3.) 24 channels<br>4.) Foire-3000, Shimadzu Co., Kyoto, Japan (CW)	1.) EEG-system<br>2.) 3.0 cm<br>3.) sitting (0-2 s before trial onset)<br>4.) HPF at 0.04 Hz; LPF at 0.7 Hz<br>5.) n.a. (arbitrary unit)<br>6.) baseline correction; averaging<br>7.) mean of oxyHb / 6-9 s after trial onset for oxyHb	1.) yes (VT)<br>2.) 11.8 ± 1.3 $\frac{mL}{kg \cdot min}$ (f) / 14.4 ± 2.7 $\frac{mL}{kg \cdot min}$ (m)<br>3.) cycling / dancing<br>4.) 10 min<br>5.) 50 % of VT<br>6.) executive functions (Stroop test)<br>7.) 5 min
**Kujach et al. [21]**	1.) 10 Hz<br>2.) 785, 830 nm<br>3.) 48 channels<br>4.) ETG-7000, Hitachi Medical Corp., Tokyo, Japan (CW)	1.) EEG-system<br>2.) 3.0 cm<br>3.) sitting (0-2 s before trial onset)<br>4.) HPF at 0.04 Hz; LPF at 0.3 Hz<br>5.) n.a. (arbitrary unit)<br>6.) baseline correction; averaging<br>7.) mean of oxyHb & deoxyHb / 4–11 s after trial onset	1.) yes ($VO_{2\,peak}$)<br>2.) 34.0 ± 5.9 $\frac{mL}{kg \cdot min}$ (f) / 157.6 ± 25.4 Watt (MAP / f) // 44.2 ± 4.9 $\frac{mL}{kg \cdot min}$ (m) / 242.2 ± 27.8 Watt (MAP / m)<br>3.) cycling<br>4.) 10 min<br>5.) 60 % of MAP<br>6.) executive functions (Stroop test)<br>7.) 15 min
**Lambrick et al. [22]**	1.) 10 Hz (exported at a sample rate of 2 Hz)<br>2.) 760, 850 nm<br>3.) 3 channel	1.) on the forehead<br>2.) 3.0, 3.5 & 4.0 cm<br>3.) sitting (10 min before CT)<br>4.) Gaussian smoothing<br>5.) constant value (4.0)<br>6.) baseline correction; averaging	1.) yes (GET / $VO_{2\,peak}$)<br>2.) 61.4 ± 8.8 $\frac{mL}{kg \cdot min}$<br>3.) treadmill running<br>4.) 15 min (continuous / intermittent in 2.5 min blocks / same group, different day)

	4.) PortaLite, Artinis Medical Systems BV, The Netherlands (CW; SRS)	7.) mean of oxyHb, deoxyHb & totHb (as proportion of the resting baseline value) / entire task period	5.) 90% of subjects GET<br>6.) executive functions (Stroop test)<br>7.) 1 min; 15 min; 30 min
**Moriya et al. [23]**	1.) 10.2 Hz<br>2.) 735, 810, 850 nm<br>3.) 2 channels<br>4.) PNIRS-10, Hamamatsu Photonics K.K., Hamamatsu, Japan (CW)	1.) EEG-system<br>2.) 3.0 cm<br>3.) sitting (10 s before CT)<br>4.) n.a.<br>5.) n.a. (arbitrary unit)<br>6.) baseline correction; averaging<br>7.) mean of oxyHb / entire task period	1.) n.a.<br>2.) n.a.<br>3.) cycling<br>4.) 15 min<br>5.) 40 % of subjects $VO_{2\,peak}$<br>6.) working memory (Sternberg task)<br>7.) 15 min
**Murata et al. [24]**	1.) 8 Hz<br>2.) 780, 805, 830 nm<br>3.) 44 channels<br>4.) OMM-3006, Shimadzu Co., Kyoto, Japan (CW)	1.) EEG-system<br>2.) 3.0 cm<br>3.) sitting (n.a.)<br>4.) n.a.<br>5.) n.a. (arbitrary unit)<br>6.) baseline correction; GLM<br>7.) mean of oxyHb / 6-s delayed boxcar function convolved with a Gaussian kernel of dispersion of 6-s full-width at half-maximum	1.) no (Karvonen formula)<br>2.) n.a.<br>3.) cycling<br>4.) 20 min<br>5.) 50% of subjects $VO_{2\,peak}$<br>6.) executive functions (Go/No-Go-Task)<br>7.) n.a.
**Ochi et al. [25]**	1.) 10 Hz<br>2.) 785, 830 nm<br>3.) 48 channels<br>4.) ETG-7000, Hitachi Medical Corp., Tokyo, Japan (CW)	1.) EEG-system<br>2.) 3.0 cm<br>3.) sitting (0-2 s before trial onset)<br>4.) HPF at 0.04 Hz; LPF at 0.3 Hz<br>5.) n.a. (arbitrary unit)<br>6.) baseline correction; averaging<br>7.) mean of oxyHb & deoxyHb / 6–10 s after trial onset	1.) yes ($VO_{2\,peak}$)<br>2.) 38.8 ± 6.4 $\frac{mL}{kg \cdot min}$ / 185.4 ± 47.1 Watt<br>3.) cycling (under normobaric hypoxic conditions)<br>4.) 10 min<br>5.) 50 % of $VO_{2\,peak}$<br>6.) executive functions (Stroop test)<br>7.) 15 min
**Sudo et al. [26]**	1.) 1 Hz<br>2.) 775, 810, 850 nm<br>3.) 2 channel<br>4.) NIRO-200, Hamamatsu Photonics K.K., Hamamatsu, Japan (CW; SRS)	1.) EEG-system<br>2.) 3.0 & 4.0 cm<br>3.) sitting (30 s before CT)<br>4.) n.a.<br>5.) age-dependent value (4.99 + 0.067 × age $^{0.814}$)<br>6.) baseline correction; averaging	1.) n.a.<br>2.) n.a.<br>3.) stretching vs. resting<br>4.) 30 min<br>5.) n.a.<br>6.) visual search task<br>7.) 5 min

		7.) mean of oxyHb, deoxyHb & TOI (= oxyHb/totHb × 100) / entire task period	
**Sudo et al. [27]**	1.) 10 Hz<br>2.) 780, 810, 830 nm<br>3.) 2 channel<br>4.) BOM-L1 TRW, Omegawave, Tokyo, Japan (CW; SRS)	1.) EEG-system<br>2.) 2.0 cm (SDS) & 4.0 cm<br>3.) sitting (30 s before CT)<br>4.) removal of motion artefacts, visual inspection<br>5.) constant value (4.0) / (arbitrary unit)<br>6.) baseline correction; averaging; use of short-separation channel; correlation analysis with skin blood flow<br>7.) mean of oxyHb, deoxyHb & totHb; TOI (= oxyHb/totHb × 100) / entire task period	1.) yes ($GET\ /\ VO_{2\,peak}$)<br>2.) 48.2 ± 6.6 $\frac{mL}{kg \cdot min}$ (exercise group) / 47.7 ± 7.4 $\frac{mL}{kg \cdot min}$ (control group)<br>3.) cycling<br>4.) average of ca. 16 min<br>5.) maximal exhaustion<br>6.) executive functions / working memory (Go/No-Go-task / Spatial Delayed Response-task)<br>7.) 2 min
**Tsuchiya et al. [28]**	1.) 17 Hz (60 ms)<br>2.) 780, 805, 830 nm<br>3.) 45 channels<br>4.) LABNIRS, Shimadzu Co., Kyoto, Japan (CW)	1.) EEG-system<br>2.) 1.5 cm (SDS) & 3.0 cm<br>3.) sitting (5 s before CT)<br>4.) moving average<br>5.) n.a. (arbitrary unit)<br>6.) baseline correction; use of short-separation channel (segment-independent component analysis); averaging<br>7.) mean of oxyHb / entire task period	1.) n.a.<br>2.) n.a.<br>3.) housework activities (HA; e.g. vacuuming)<br>4.) 10 min<br>5.) 19.35 ± 7.89 of % HRR (CC) / 20.47 ± 7.86 of % HRR (HA) (calculated using the Karvonen Formula)<br>6.) executive functions (Stroop test)<br>7.) 5 min
**Tsujii et al. [29]**	1.) 10.2 Hz<br>2.) 735, 810, 850 nm<br>3.) 2 channels<br>4.) PNIRS-10, Hamamatsu Photonics K.K., Hamamatsu, Japan (CW; SRS)	1.) EEG-system<br>2.) 3.0 cm<br>3.) sitting (10 s before CT)<br>4.) n.a.<br>5.) n.a. (arbitrary unit)<br>6.) baseline correction; averaging<br>7.) mean of oxyHb / entire task period	1.) yes (estimated $VO_{2\,peak}$)<br>2.) n.a.<br>3.) cycling<br>4.) 10 min<br>5.) 40 % of subjects $VO_{2\,peak}$<br>6.) working memory (Sternberg task)<br>7.) 10 min
**Yamazaki et al. [30]**	1.) 17 Hz (60 ms)<br>2.) 780, 805, 830 nm<br>3.) 39 channels	1.) EEG-system / individual MRI scan<br>2.) 3.0 cm<br>3.) recumbent sitting (120 s before CT)<br>4.) LPF at 0.1 Hz	1.) yes ($VO_{2\,peak}$)<br>2.) 36.0 ± 5.8 $\frac{mL}{kg \cdot min}$<br>3.) recumbent cycling

	4.) LABNIRS, Shimadzu Co., Kyoto, Japan (CW)	5.) n.a. (arbitrary unit)<br>6.) baseline correction; averaging<br>7.) mean of oxyHb / 2 s before trial onset to 13.5 s after trial onset	4.) 10 min<br>5.) 30 % of subjects $VO_{2\,peak}$<br>6.) spatial working memory<br>7.) 5 min; 30 min
**Yanagisawa et al. [31]**	1.) 10 Hz<br>2.) 785, 830 nm<br>3.) 48 channels<br>4.) ETG-7000, Hitachi Medical Corp., Tokyo, Japan (CW)	1.) EEG-system<br>2.) 3.0 cm<br>3.) sitting (0-2 s before trial onset)<br>4.) HPF at 0.04 Hz; LPF at 0.7 Hz<br>5.) n.a. (arbitrary unit)<br>6.) baseline correction; averaging; visual inspection<br>7.) mean of oxyHb & deoxyHb / 4–11 s after trial onset	1.) yes ($VO_{2\,peak}$)<br>2.) 46.3 ± 10.4 $\frac{mL}{kg \cdot min}$<br>3.) cycling<br>4.) 10 min<br>5.) 50% of a subject's $VO_{2\,peak}$<br>6.) executive functions (Stroop test)<br>7.) 15 min
**Studies conducting long-term physical exercises**			
**Chen et al. [32]**	1.) 10 Hz<br>2.) 695, 830 nm<br>3.) 44 channels<br>4.) ETG-4000, Hitachi Medical Corp., Tokyo, Japan (CW)	1.) EEG-system<br>2.) 3.0 cm<br>3.) sitting (30 s before CT)<br>4.) n.a.<br>5.) n.a. (arbitrary unit)<br>6.) baseline correction; averaging<br>7.) mean of oxyHb & deoxyHb / entire task period	1.) no<br>2.) n.a.<br>3.) Baduanjin Mind-Body Intervention (BMB) / Muscle Relaxation<br>4.) 90 min // 5 times per week; 8 weeks long<br>5.) n.a.<br>6.) executive functions (Flanker test)<br>7.) n.a.
**Coetsee et al. [33]**	1.) 5 Hz<br>2.) 735, 810, 850 nm<br>3.) 2 channels<br>4.) NIRO 200NX, Hamamatsu Photonics K.K., Hamamatsu, Japan (CW; SRS)	1.) EEG-system<br>2.) 4.0 cm<br>3.) sitting (5 min before CT, eyes closed)<br>4.) n.a.<br>5.) constant value (5.9) / n.a (THI)<br>6.) baseline correction; averaging<br>7.) mean of oxyHb, deoxyHb & totHb / entire task period	1.) yes (MCT & HIIT; calculated HR_{max} = 220 – age) / yes (ReT; 10 RM)<br>2.) 19 $\frac{mL}{kg \cdot min}$ (determined by submaximal Bruce treadmill test)<br>3.) ReT, MCT & HIIT (walking)<br>4.) 30 min (ReT & HIIT) / 47 min (MCT) // 3 times per week, 16 weeks long<br>5.) increasing load (ReT); 70–75% HR_{max} (MCT); 90–95% HR_{max} (HIIT)<br>6.) executive functions (Stroop test)<br>7.) n.a.
**Wang et al. [34]**	1.) 10 Hz<br>2.) 760, 840 nm<br>3.) 24 channels	1.) EEG-system<br>2.) 3.0 cm<br>3.) sitting (10 s before / 25 s after CT)	1.) no<br>2.) n.a.<br>3.) Tai-Chi exercise

	4.) ETG-100, Hitachi Medical Corp., Tokyo, Japan (CW)	4.) 5 s moving average; manual removal of motion artefacts; linear fitting<br>5.) n.a. (arbitrary unit)<br>6.) baseline correction; averaging; visual inspection<br>7.) mean of oxyHb / entire task period	4.) 100 min // one a week, 6 months long<br>5.) n.a.<br>6.) executive functions (Stroop test)<br>7.) n.a.
**Xu et al. [35]**	1.) 7.81 Hz<br>2.) 760, 850 nm<br>3.) 20 channels<br>4.) NIRScout, NIRx Medical Technologies LLC, USA (CW)	1.) EEG-system<br>2.) 3.0 cm<br>3.) sitting<br>4.) HPF at 0.01 Hz; LPF at 0.3 Hz<br>5.) n.a. (arbitrary unit)<br>6.) baseline correction; averaging<br>7.) mean of oxyHb / 12 s time period	1.) no<br>2.) n.a.<br>3.) aerobic and anaerobic exercises<br>4.) 90 min – 150 min // 3 times per day, 4 weeks long<br>5.) n.a.<br>6.) executive functions (Stroop test)<br>7.) n.a.
**Cross-sectional studies**			
**Albinet et al. [36]**	1.) 6 Hz<br>2.) 775, 810, 850 nm<br>3.) 2 channels<br>4.) NIRO-200, Hamamatsu Photonics K.K., Hamamatsu, Japan (CW; SRS)	1.) EEG-system<br>2.) 4.0 cm<br>3.) sitting (last 10 s of 100 s before CT)<br>4.) LPF at 0.7 Hz, slope method<br>5.) n.a. (arbitrary unit)<br>6.) baseline correction; averaging; visual inspection<br>7.) mean of oxyHb & deoxyHb / last 10 s of task period for regular statistical analysis and 100-s stimulation windows for slope method analysis	1.) yes ($VO_{2\,peak}$)<br>2.) 20 ± 2.7 $\frac{mL}{kg \cdot min}$ (low-fit group) / 29.8 ± 6.5 $\frac{mL}{kg \cdot min}$ (high-fit group)<br>3.) n.a.<br>4.) n.a.<br>5.) n.a.<br>6.) executive functions (Random Number Generation)<br>7.) n.a.
**Cameron et al. [37]**	1.) 1 Hz<br>2.) 775, 810, 850 nm<br>3.) 1 channel<br>4.) NIRO-200, Hamamatsu Photonics K.K., Hamamatsu, Japan (CW; SRS)	1.) EEG-system<br>2.) 4.0 cm<br>3.) sitting (2 min before CT)<br>4.) n.a.<br>5.) constant value (5.93)<br>6.) baseline correction; averaging; visual inspection<br>7.) median of oxyHb, deoxyHb & totHb / entire task period	1.) no<br>2.) n.a. (physical activity questionnaire)<br>3.) n.a.<br>4.) n.a.<br>5.) n.a.<br>6.) executive functions (reaction time task; inhibitory control)<br>7.) n.a.

**Dupuy et al. [38]**	1.) 10 Hz<br>2.) 690, 830 nm<br>3.) 14 channels<br>4.) CW6, TechEn Inc., Milford, MA, USA (CW)	1.) EEG-system<br>2.) 2.8 cm<br>3.) sitting (1 min before CT)<br>4.) PCA; HPF at 0.0042 Hz; HRF<br>5.) constant value (5.93)<br>6.) baseline correction; averaging<br>7.) mean of oxyHb, deoxyHb &; totHb / entire task period	1.) yes ($VO_{2\,peak}$)<br>2.) ▪ 43.8 ± 8.0 $\frac{mL}{kg \cdot min}$ younger adults<br>- 36.4 ± 5.3 $\frac{mL}{kg \cdot min}$ (low-fit younger adults)<br>- 46.6 ± 7.0 $\frac{mL}{kg \cdot min}$ (high-fit younger adults)<br>▪ 28.7 ± 7.3 $\frac{mL}{kg \cdot min}$ older adults<br>- 21.4 ± 7.1 $\frac{mL}{kg \cdot min}$ (low-fit older adults)<br>- 30.1 ± 1.5 $\frac{mL}{kg \cdot min}$ (high-fit older adults)<br>3.) n.a.<br>4.) n.a.<br>5.) n.a.<br>6.) executive functions (Stroop test)<br>7.) n.a
**Fabiani et al. [39]**	1.) 0.625 Hz<br>2.) 690, 830 nm<br>3.) 80 channels<br>4.) Imagent device, ISS Inc., Champaign, IL, USA (FD)	1.) on occipital cortex (MRI co-registration)<br>2.) 2.0 to 5.0 cm<br>3.) sitting (10 s before stimulation)<br>4.) n.a.<br>5.) n.a. (frequency-domain fNIRS)<br>6.) baseline correction; averaging;<br>7.) mean of oxyHb & deoxyHb / 5-19.2 s after the onset of stimulation	1.) yes ($VO_{2\,peak}$)<br>2.) 30.7 ± 6.7 $\frac{mL}{kg \cdot min}$ (high-fit older adults) /<br>18.9 ± 3.8 $\frac{mL}{kg \cdot min}$ (low-fit older adults)<br>3.) n.a.<br>4.) n.a.<br>5.) n.a.<br>6.) visual stimulation (checkerboard reversals at different frequencies)<br>7.) n.a
**Giles et al. [40]**	1.) 7.81 Hz<br>2.) 760, 850 nm<br>3.) 21 channels<br>4.) NIRSport, NIRx Medical Technologies, LLC, New York, USA (CW)	1.) EEG-system<br>2.) 3.0 cm<br>3.) sitting<br>4.) HPF at 0.01 Hz; LPF at 0.2 Hz; spike artefact removal, visual inspection<br>5.) 7.25 / 6.38 (760 nm / 850 nm)<br>6.) baseline correction; averaging<br>7.) mean of oxyHb, deoxyHb & totHb / first 4 s of a trial for preparatory	1.) no<br>2.) n.a. (physical activity questionnaire)<br>3.) n.a.<br>4.) n.a.<br>5.) n.a.<br>6.) executive functions / control of emotion (Stroop test / cognitive reappraisal task)<br>7.) n.a.

		period and 4 – 12 after trial onset for regulatory period	
**Hyodo et al. [41]**	1.) 10 Hz<br>2.) 785, 830 nm<br>3.) 48 channels<br>4.) ETG-7000, Hitachi Medical Corp., Tokyo, Japan (CW)	1.) EEG-system<br>2.) 3.0 cm<br>3.) sitting (0-2 s before trial onset)<br>4.) HPF at 0.04 Hz; LPF at 0.7 Hz<br>5.) n.a. (arbitrary unit)<br>6.) baseline correction; averaging<br>7.) mean of oxyHb & deoxyHb / 6–9 s after trial onset	1.) yes (VT)<br>2.) 14.9 ± 3.8 $\frac{mL}{kg \cdot min}$ (VT)<br>3.) n.a.<br>4.) n.a.<br>5.) n.a.<br>6.) executive functions (Stroop test)<br>7.) n.a.
**Kato et al. [42]**	1.) 10 Hz<br>2.) 780, 805, 830 nm<br>3.) 22 channel<br>4.) Foire-3000, Shimadzu Co., Kyoto, Japan (CW)	1.) EEG-system<br>2.) 3.0 cm<br>3.) sitting (30 s before CT)<br>4.) n.a.<br>5.) n.a. (arbitrary unit)<br>6.) averaging; visual inspection<br>7.) peak of oxyHb & AUC of oxyHb / time to peak	1.) no<br>2.) n.a. (physical activity tracking via actigraphy)<br>3.) n.a.<br>4.) n.a.<br>5.) n.a.<br>6.) executive functions / memory (verbal fluency task)<br>7.) n.a.
**Makizako et al. [43]**	1.) 1.54 Hz<br>2.) 770, 840 nm<br>3.) 16 channels<br>4.) OEG-16 system, Spectratech Inc., Yokohama, Japan (CW)	1.) EEG-system<br>2.) 3.0 cm<br>3.) sitting (10 s before / after CT)<br>4.) LPF at 0.05 Hz<br>5.) n.a. (arbitrary unit)<br>6.) baseline correction; averaging<br>7.) mean of oxyHb / entire task period	1.) no<br>2.) n.a. (physical activity questionnaire)<br>3.) n.a.<br>4.) n.a.<br>5.) n.a.<br>6.) executive functions / memory (verbal fluency task)<br>7.) n.a.
**Matsuda et al. [44]**	1.) 10 Hz<br>2.) 695, 830 nm<br>3.) 32 channels<br>4.) ETG-4000, Hitachi Medical Corp., Tokyo, Japan (CW)	1.) EEG-system<br>2.) 3.0 cm<br>3.) sitting (2 s before CT)<br>4.) n.a.<br>5.) n.a. (arbitrary unit)<br>6.) baseline correction; averaging<br>7.) mean of oxyHb / 4–11 s after trial onset	1.) no<br>2.) n.a. (physical activity tracking via accelerometer / questionnaire)<br>3.) n.a.<br>4.) n.a.<br>5.) n.a.<br>6.) executive functions (Stroop test)<br>7.) n.a.
**Mücke et al. [45]**	1.) 7.81 Hz<br>2.) 760, 850 nm<br>3.) 16 channels	1.) EEG-system<br>2.) 2.7 to 3.4 cm<br>3.) sitting (2 min before CT)	1.) no<br>2.) 94.4 ± 26.0 $\frac{min}{day}$ low MVPA / 161.6 ± 27.0 $\frac{min}{day}$ high MVPA

	4.) NIRSport, NIRx Medizintechnik GmbH, Berlin, Germany (CW)	4.) discontinuities with more than 5 SD were removed; Spike artefact correction; HPF at 0.01 Hz; LPF at 0.2 Hz; noisy channels with more than 2.5 SD were removed<br>5.) 7.25 / 6.38 (760 nm / 850 nm)<br>6.) baseline correction; averaging<br>7.) mean of oxyHb / entire task period	3.) n.a.<br>4.) n.a.<br>5.) n.a.<br>6.) executive functions (semantic and phonetic verbal fluency tests; mental arithmetic)<br>7.) n.a.
**Suhr & Chellenberg [46]**	1.) 15 Hz<br>2.) 710, 830 nm<br>3.) 4 channels<br>4.) INVOS™ 5100, Somanetics Corporation, Troy, MI, USA (SRS)	1.) on the forehead<br>2.) 3.0 & 4.0 cm<br>3.) sitting (90 s before CT)<br>4.) n.a.<br>5.) n.a. (rSO_2)<br>6.) baseline correction; averaging<br>7.) mean of rSO_2 / 90 s prior onset of cognitive testing	1.) no<br>2.) n.a. (physical activity questionnaire)<br>3.) n.a.<br>4.) n.a.<br>5.) n.a.<br>6.) cognitive test battery<br>7.) n.a.

AUC: area und the curve; BMB: Baduanjin Mind-Body Intervention; CC: control condition; cm: centimeter; CT: cognitive testing; CW: continuous-wave near-infrared spectroscopy; deoxyHb: deoxygenated hemoglobin; DPF: differential path length factor; EEG: electroencephalography; Ex_{max}: maximal exercise intensity (in watt); f: female; FD: frequency domain near-infrared spectroscopy; GET: gaseous exchange threshold; GLM: general linear model; GXT: graded exercise test; HC: healthy controls; HIA: high-intensity aerobic exercise; HIIT: high-intensity aerobic interval training; HIR; high-intensity resistance training; HP: high performer; HPF: high-pass filter; HRF: hemodynamic response function; HR_{max}: maximal heart rate; HRR: heart rate reserve; Hz: Hertz; kg: kilogram; LP: low performer; LPF: low-pass filter; m: male; MAP: maximal aerobic power; MCT: moderate continuous aerobic training; MIC: moderate-intensity exercise combining resistance training and walking; min: minute; ml: milliliter; MRI: magnetic resonance imaging; ms: milliseconds; MVPA: moderate-to-vigorous physical activity; n.a.: not applicable; nm: nanometer; oxyHb: oxygenated hemoglobin; PCA: principle component analysis; PP: peak performance in exercise test; RM: repetition maximum; ROI: region of interest; ReT: resistance training; s: second; SD: standard deviation; SDS: short-distance separation; SRS: spatially resolved near-infrared spectroscopy; TIA: patients with transient ischemic attack; TOI (or rSO_2): tissue oxygenation index; totHb: total hemoglobin; vs.: versus; $VO_{2\,peak}$: maximal oxygen uptake; VT: ventilatory threshold; W: watt.

Supplementary figure

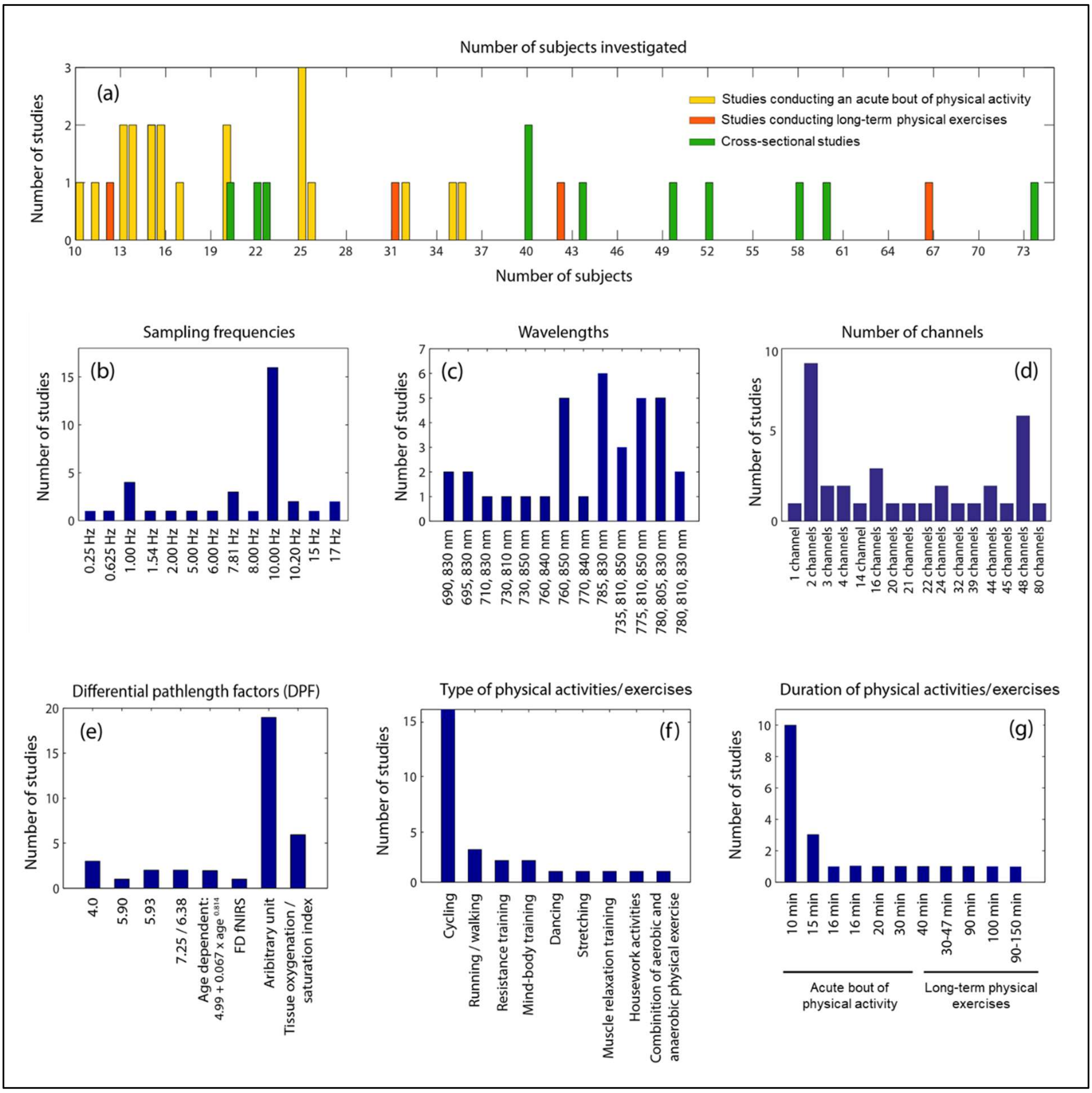

Figure S1. Overview on (a) number of subjects investigated, (b) sampling frequencies (c) wavelengths, (d) number of channels, (e) differential pathlength factors (DPF), (f) types of physical activities / exercises and (g) durations of physical activities / exercises.

References

1. Scholkmann, F.; Kleiser, S.; Metz, A.J.; Zimmermann, R.; Mata Pavia, J.; Wolf, U.; Wolf, M. A review on continuous wave functional near-infrared spectroscopy and imaging instrumentation and methodology. NeuroImage 2014, 85, 6–27, doi:10.1016/j.neuroimage.2013.05.004.
2. Rupawala, M.; Dehghani, H.; Lucas, S.J.E.; Tino, P.; Cruse, D. Shining a Light on Awareness: A Review of Functional Near-Infrared Spectroscopy for Prolonged Disorders of Consciousness. Front. Neurol. 2018, 9, 68, doi:10.3389/fneur.2018.00350.
3. Gervain, J.; Mehler, J.; Werker, J.F.; Nelson, C.A.; Csibra, G.; Lloyd-Fox, S.; Shukla, M.; Aslin, R.N. Near-infrared spectroscopy: a report from the McDonnell infant methodology consortium. Developmental cognitive neuroscience 2011, 1, 22–46, doi:10.1016/j.dcn.2010.07.004.
4. Scholkmann, F.; Wolf, M. Measuring brain activity using functional near infrared spectroscopy: a short review. Spectroscopy Europe 2012, 24, 6–10.
5. Delpy, D.T.; Cope, M. Quantification in tissue near-infrared spectroscopy. Philosophical Transactions of the Royal Society B: Biological Sciences 1997, 352, 649–659, doi:10.1098/rstb.1997.0046.
6. Scholkmann, F.; Metz, A.J.; Wolf, M. Measuring tissue hemodynamics and oxygenation by continuous-wave functional near-infrared spectroscopy—how robust are the different calculation methods against movement artifacts? Physiological measurement 2014, 35, 717–734, doi:10.1088/0967-3334/35/4/717.
7. Kohl-Bareis, M. NIRS: Theoretical Background and Practical Aspects. In Functional Neuroimaging in Exercise and Sport Sciences; Boecker, H., Hillman, C.H., Scheef, L., Strüder, H.K., Eds.; Springer New York: New York, NY, 2012; pp 213–235.
8. Hoshi, Y. Functional Near-Infrared Spectroscopy: Potential and Limitations in Neuroimaging Studies. Neuroimaging, Part A; Elsevier, 2005; pp 237–266.
9. Hoshi, Y.; Yamada, Y. Overview of diffuse optical tomography and its clinical applications. Journal of biomedical optics 2016, 21, 91312, doi:10.1117/1.JBO.21.9.091312.
10. Elwell, C.E.; Cooper, C.E. Making light work: Illuminating the future of biomedical optics. Philosophical transactions. Series A, Mathematical, physical, and engineering sciences 2011, 369, 4358–4379, doi:10.1098/rsta.2011.0302.
11. Ferrari, M.; Quaresima, V. A brief review on the history of human functional near-infrared spectroscopy (fNIRS) development and fields of application. NeuroImage 2012, 63, 921–935, doi:10.1016/j.neuroimage.2012.03.049.
12. Ando, S.; Yamada, Y.; Kokubu, M. Reaction time to peripheral visual stimuli during exercise under hypoxia. Journal of applied physiology (Bethesda, Md. : 1985) 2010, 108, 1210–1216, doi:10.1152/japplphysiol.01115.2009.
13. Bediz, C.S.; Oniz, A.; Guducu, C.; Ural Demirci, E.; Ogut, H.; Gunay, E.; Cetinkaya, C.; Ozgoren, M. Acute Supramaximal Exercise Increases the Brain Oxygenation in Relation to Cognitive Workload. Front. Hum. Neurosci. 2016, 10, 771, doi:10.3389/fnhum.2016.00174.
14. Byun, K.; Hyodo, K.; Suwabe, K.; Ochi, G.; Sakairi, Y.; Kato, M.; Dan, I.; Soya, H. Positive effect of acute mild exercise on executive function via arousal-related prefrontal activations: an fNIRS study. NeuroImage 2014, 98, 336–345, doi:10.1016/j.neuroimage.2014.04.067.
15. Chang, H.; Kim, K.; Jung, Y.-J.; Kato, M. Effects of Acute High-Intensity Resistance Exercise on Cognitive Function and Oxygenation in Prefrontal Cortex. Journal of exercise nutrition & biochemistry 2017, 21, 1–8, doi:10.20463/jenb.2017.0012.
16. Endo, K.; Matsukawa, K.; Liang, N.; Nakatsuka, C.; Tsuchimochi, H.; Okamura, H.; Hamaoka, T. Dynamic exercise improves cognitive function in association with increased prefrontal oxygenation. The journal of physiological sciences : JPS 2013, 63, 287–298, doi:10.1007/s12576-013-0267-6.
17. Faulkner, J.; Lambrick, D.; Kaufmann, S.; Stoner, L. Effects of Upright and Recumbent Cycling on Executive Function and Prefrontal Cortex Oxygenation in Young, Healthy, Men. Journal of physical activity & health 2016, doi:10.1123/jpah.2015-0454.
18. Faulkner, J.; Stoner, L.; Grigg, R.; Fryer, S.; Stone, K.; Lambrick, D. Acute effects of exercise posture on executive function in transient ischemic attack patients. Psychophysiology 2017, doi:10.1111/psyp.12868.
19. Hyodo, K.; Dan, I.; Suwabe, K.; Kyutoku, Y.; Yamada, Y.; Akahori, M.; Byun, K.; Kato, M.; Soya, H. Acute moderate exercise enhances compensatory brain activation in older adults. Neurobiology of Aging 2012, 33, 2621–2632, doi:10.1016/j.neurobiolaging.2011.12.022.

20. Hyodo, K.; Suwabe, K.; Soya, H.; Nagamatsu, T. The effect of an acute bout of slow aerobic dance on mood and executive function in older adults: a pilot study. Bulletin of the Physical Fitness Research Institute 2017, 35–41.
21. Kujach, S.; Byun, K.; Hyodo, K.; Suwabe, K.; Fukuie, T.; Laskowski, R.; Dan, I.; Soya, H. A transferable high-intensity intermittent exercise improves executive performance in association with dorsolateral prefrontal activation in young adults. NeuroImage 2017, doi:10.1016/j.neuroimage.2017.12.003.
22. Lambrick, D.; Stoner, L.; Grigg, R.; Faulkner, J. Effects of continuous and intermittent exercise on executive function in children aged 8-10 years. Psychophysiology 2016, doi:10.1111/psyp.12688.
23. Moriya, M.; Aoki, C.; Sakatani, K. Effects of Physical Exercise on Working Memory and Prefrontal Cortex Function in Post-Stroke Patients. Advances in experimental medicine and biology 2016, 923, 203–208, doi:10.1007/978-3-319-38810-6_27.
24. Murata, Y.; Watanabe, A.; Terasawa, S.; Nakajima, K.; Kobayashi, T.; Yong, Z.; Okuhara, M.; Nakade, K.; Terasawa, K.; Maruo, S.K. Moderate Exercise Improves Cognitive Performance and Decreases Cortical Activation in Go/No-Go Task. BAOJMN 2015, 1, 1–7, doi:10.24947/baojmn/1/1/102.
25. Ochi, G.; Yamada, Y.; Hyodo, K.; Suwabe, K.; Fukuie, T.; Byun, K.; Dan, I.; Soya, H. Neural basis for reduced executive performance with hypoxic exercise. NeuroImage 2018, 171, 75–83, doi:10.1016/j.neuroimage.2017.12.091.
26. Sudo, M.; Ando, S.; Nagamatsu, T. Effects of acute static stretching on visual search performance and mood state. JPES 2015, 15, 651–656, doi:10.7752/jpes.2015.04099.
27. Sudo, M.; Komiyama, T.; Aoyagi, R.; Nagamatsu, T.; Higaki, Y.; Ando, S. Executive function after exhaustive exercise. European journal of applied physiology 2017, doi:10.1007/s00421-017-3692-z.
28. Tsuchiya, K.; Mitsui, S.; Fukuyama, R.; Yamaya, N.; Fujita, T.; Shimoda, K.; Tozato, F. An acute bout of housework activities has beneficial effects on executive function. Neuropsychiatric disease and treatment 2018, 14, 61–72, doi:10.2147/NDT.S153813.
29. Tsujii, T.; Komatsu, K.; Sakatani, K. Acute effects of physical exercise on prefrontal cortex activity in older adults: A functional near-infrared spectroscopy study. Advances in experimental medicine and biology 2013, 765, 293–298, doi:10.1007/978-1-4614-4989-8_41.
30. Yamazaki, Y.; Sato, D.; Yamashiro, K.; Tsubaki, A.; Yamaguchi, Y.; Takehara, N.; Maruyama, A. Inter-individual Differences in Exercise-Induced Spatial Working Memory Improvement: A Near-Infrared Spectroscopy Study. Advances in experimental medicine and biology 2017, 977, 81–88, doi:10.1007/978-3-319-55231-6_12.
31. Yanagisawa, H.; Dan, I.; Tsuzuki, D.; Kato, M.; Okamoto, M.; Kyutoku, Y.; Soya, H. Acute moderate exercise elicits increased dorsolateral prefrontal activation and improves cognitive performance with Stroop test. NeuroImage 2010, 50, 1702–1710, doi:10.1016/j.neuroimage.2009.12.023.
32. Chen, T.; Yue, G.H.; Tian, Y.; Jiang, C. Baduanjin Mind-Body Intervention Improves the Executive Control Function. Frontiers in psychology 2016, 7, 2015, doi:10.3389/fpsyg.2016.02015.
33. Coetsee, C.; Terblanche, E. Cerebral oxygenation during cortical activation: The differential influence of three exercise training modalities. A randomized controlled trial. European journal of applied physiology 2017, doi:10.1007/s00421-017-3651-8.
34. Wang, W.; Qiu, C.; Ota, T.; Sawada, M.; Kishimoto, N.; Kishimoto, T. Effects of Tai-Chi Exercise on Attention in Healthy Elderly Subject as measured by Near-Infrared Spectroscopy during the Stroop Task. J. Nara Med. Assoc. (Journal of Nara Medical Association) 2013, 64, 79–86.
35. Xu, X.; Deng, Z.-Y.; Huang, Q.; Zhang, W.-X.; Qi, C.-Z.; Huang, J.-A. Prefrontal cortex-mediated executive function as assessed by Stroop task performance associates with weight loss among overweight and obese adolescents and young adults. Behavioural Brain Research 2017, 321, 240–248, doi:10.1016/j.bbr.2016.12.040.
36. Albinet, C.T.; Mandrick, K.; Bernard, P.L.; Perrey, S.; Blain, H. Improved cerebral oxygenation response and executive performance as a function of cardiorespiratory fitness in older women: a fNIRS study. Front. Aging Neurosci. 2014, 6, 273185, doi:10.3389/fnagi.2014.00272.
37. Cameron, T.A.; Lucas, S.J.E.; Machado, L. Near-infrared spectroscopy reveals link between chronic physical activity and anterior frontal oxygenated hemoglobin in healthy young women. Psychophysiology 2015, 52, 609–617, doi:10.1111/psyp.12394.
38. Dupuy, O.; Gauthier, C.J.; Fraser, S.A.; Desjardins-Crepeau, L.; Desjardins, M.; Mekary, S.; Lesage, F.; Hoge, R.D.; Pouliot, P.; Bherer, L. Higher levels of cardiovascular fitness are associated with better executive function

and prefrontal oxygenation in younger and older women. Frontiers in human neuroscience 2015, 9, 66, doi:10.3389/fnhum.2015.00066.
39. Fabiani, M.; Gordon, B.A.; Maclin, E.L.; Pearson, M.A.; Brumback-Peltz, C.R.; Low, K.A.; McAuley, E.; Sutton, B.P.; Kramer, A.F.; Gratton, G. Neurovascular coupling in normal aging: a combined optical, ERP and fMRI study. NeuroImage 2014, 85 Pt 1, 592–607, doi:10.1016/j.neuroimage.2013.04.113.
40. Giles, G.E.; Cantelon, J.A.; Eddy, M.D.; Brunyé, T.T.; Urry, H.L.; Mahoney, C.R.; Kanarek, R.B. Habitual exercise is associated with cognitive control and cognitive reappraisal success. Experimental Brain Research 2017, doi:10.1007/s00221-017-5098-x.
41. Hyodo, K.; Dan, I.; Kyutoku, Y.; Suwabe, K.; Byun, K.; Ochi, G.; Kato, M.; Soya, H. The association between aerobic fitness and cognitive function in older men mediated by frontal lateralization. NeuroImage 2016, 125, 291–300, doi:10.1016/j.neuroimage.2015.09.062.
42. Kato, K.; Iwamoto, K.; Kawano, N.; Noda, Y.; Ozaki, N.; Noda, A. Differential effects of physical activity and sleep duration on cognitive function in young adults. Journal of Sport and Health Science 2017, doi:10.1016/j.jshs.2017.01.005.
43. Makizako, H.; Doi, T.; Shimada, H.; Park, H.; Uemura, K.; Yoshida, D.; Tsutsumimoto, K.; Anan, Y.; Suzuki, T. Relationship between going outdoors daily and activation of the prefrontal cortex during verbal fluency tasks (VFTs) among older adults: A near-infrared spectroscopy study. Archives of Gerontology and Geriatrics 2013, 56, 118–123, doi:10.1016/j.archger.2012.08.017.
44. Matsuda, K.; Ikeda, S.; Mitsutake, T.; Nakahara, M.; Nagai, Y.; Ikeda, T.; Horikawa, E. Factors influencing executive function by physical activity level among young adults: A near-infrared spectroscopy study. Journal of physical therapy science 2017, 29, 470–475, doi:10.1589/jpts.29.470.
45. Mücke, M.; Andra, C.; Gerber, M.; Puhse, U.; Ludyga, S. Moderate-to-vigorous physical activity, executive functions and prefrontal brain oxygenation in children: A functional near-infrared spectroscopy study. Journal of sports sciences 2017, 1–7, doi:10.1080/02640414.2017.1326619.
46. Suhr, J.A.; Chelberg, M.B. Use of near-infrared spectroscopy as a measure of cerebrovascular health in aging adults. Neuropsychology, development, and cognition. Section B, Aging, neuropsychology and cognition 2013, 20, 243–252, doi:10.1080/13825585.2012.727976.
